# Determination of Rice Accession Status Using Infochemical and Visual Cues Emitted to Sustainably Control *Diopsis apicalis* Dalman

**DOI:** 10.3390/insects16080752

**Published:** 2025-07-23

**Authors:** Roland Bocco, Esther Pegalepo, Abou Togola, Francis Nwilene, Christophe Bernard Gandonou, Yedomon Ange Bovys Zoclanclounon, Marie Noelle Ndjiondjop, Mounirou Sow, Jeong Jun Kim, Manuele Tamò

**Affiliations:** 1 Texas AgriLife Research and Extension Center, Texas A&M University, System, 1509 Aggie Dr., Beaumont, TX 77713, USA; 2 Africa Rice Center, Bouaké 01 BP 2551, Côte d’Ivoire; e.pegalepo@cgiar.org (E.P.); m.ndjiondjop@cgiar.org (M.N.N.); 3 International Maize and Wheat Improvement Center (CIMMYT), ICRAF Campus, United Nations Avenue, Gigiri, Nairobi P.O. Box 1041-00621, Kenya; a.togola@cgiar.org; 4 AfricaRice Nigeria Country Office, c/o IITA, Ibadan PMB 5320, Oyo State, Nigeria; f.nwilene@cgiar.org; 5 Laboratoire de Physiologie Végétale et d’Etude des Stress Environnementaux, Faculté des Sciences et Techniques (FAST), Université d’Abomey-Calavi (UAC), Cotonou 06 BP 1442, Benin; ganchrist@gmail.com; 6 Plant Sciences and the Bioeconomy, Rothamsted Research, Harpenden AL5 2JQ, UK; angez9914@gmail.com; 7 Sow AgriTech Ltd., Abdulahi Fodio Road, Opposite Government Technical College, Runjin Sambo, Sokoto PMB 2999, Sokoto State, Nigeria; msow@sowagritech.com; 8 National Institute of Agricultural Science, 166 Nongsaengmyeong-ro, Iseo-myeon, Wanju-gun 55365, Jeonbuk-do, Republic of Korea; jjunkim66@gmail.com; 9 IITA-Benin, Tri Postal, Cotonou 08 BP 0932, Benin; m.tamo@cgiar.org

**Keywords:** olfactometer, stalk-eyed flies, odor, resistant variety, susceptible varieties, ovipositioning

## Abstract

This study assessed the host plant selection behavior of female stalk-eyed flies (SEFs) or *Diopsis apicalis*, where a Y-tube olfactometer was used to compare SEF attraction to the odor of leaves from four rice varieties (ITA306, WAB56-104, CG14, and RAM55). Another step of the evaluation consisted of pairing leaf odors from two rice varieties. Also, potted plants of the tested varieties were displayed in a screened cage and submitted to female SEF selection. The results indicated that the odor produced by leaves from rice varieties CG14, WAB56-104, and ITA306 significantly attracted SEFs, at rates of 81%, 70%, and 97%, respectively, while SEF females were rarely attracted by the odor of leaves from the resistant rice variety RAM55, at a rate of 35%. The results suggested that the use of a Y-tube olfactometer was similar to the use of a screened cage. The resistance exhibited by rice variety CG14 against SEFs is related to an antibiosis interaction acting as bait, while that in RAM55 is an antixenosis one. Farmers can plant the traditional CG14 variety on the edge of rice fields to draw SEFs and poison their larvae. However, RAM55 can be inserted in an intercropping system to repel SEFs from laying eggs. The authors recommend CG14 and RAM55 as candidates for breeding to create resistant lines against SEF.

## Simple Summary

This study investigated how female stalk-eyed flies (*Diopsis apicalis*), which are pests of rice crops, select specific rice plants for laying their eggs based on the plants’ scents and appearance. A Y-shaped tube was used to observe fly responses to leaf odors from four rice varieties: ITA306, WAB56-104, CG14, and RAM55. In addition, live plants were placed in cages to confirm flies’ preferences in more natural settings. The results showed that CG14, WAB56-104, and ITA306 attracted most of the flies (between 70% and 97%), while RAM55 attracted significantly fewer (only 35%). Although CG14 attracted many flies for egg-laying, it negatively affected the development of their larvae, thereby reducing overall damage. In contrast, RAM55 deterred them from laying eggs. These findings offer new tools for protecting rice: CG14 can be planted around vulnerable crops to trap and weaken pests, and RAM55 can be mixed into fields to keep insects away. The study also helped scientists understand how plants resist pests, making CG14 and RAM55 strong candidates for breeding new rice varieties that are better defended against insect attacks.

## 1. Introduction

Rice is an important cereal, after wheat, and is utilized as a staple food in numerous African countries and worldwide, ultimately contributing to food security [1]. Nigeria, Mali, Tanzania, and Ghana are among the leading rice producers in Sub-Saharan Africa (SSA). Nigeria is the largest producer, supplying around 8.5 million metric tons per year [2]. Mali and Tanzania are other prominent growers, each contributing significantly to the region’s rice production [2]. Despite increased rice production, SSA still imports over one-third of its rice, accounting for 40% of global rice exports. Asia (especially Thailand, Vietnam, Pakistan, and India) is the main source of these imports [3]. Rice cultivation in the region is characterized by different ecosystems, such as rainfed upland, rainfed lowland, and irrigated systems [4]. Despite rising demand due to population increase, urbanization, and shifting dietary habits, local supply covers only approximately 60% of demand, necessitating significant imports [5]. This reliance on imports, which costs more than $6 billion per year, highlights the economic need to increase local rice production [6]. Many abiotic and biotic challenges exist in this zone for rice farming.

The dipterous insects that affect cultivated rice are among the main stem borer species in the wet areas of West Africa, and female stalk-eyed flies (SEFs), or *Diopsis apicalis* Dalman, are particularly common and harmful in this part of the continent [7]. This insect causes severe attacks on rice in West African countries, where stem damage leads to high yield loss. This pest is reported in all rice ecological zones of tropical Africa [8], but preferentially in wet and shady lowland areas [9,10] and also in irrigated rice fields [11].

Historically, SEF control in African rice farming systems has relied exclusively on the use of chemical insecticides [12]. However, these pesticides alone rarely provide long-term solutions. In contrast, their use affects not only applicators, consumers, and pests, but also natural enemies and the environment [13]. Furthermore, inappropriate chemical use can create outbreaks of secondary pests and resistant pest strains. Nowadays, a more sustainable pest management approach includes all compatible control options, such as biological control and the use of resistant or tolerant varieties. Togola et al. [7] observed that upland NERICA (New Rice for Africa) rice varieties inherited SEF resistance from their donor CG14. For decades, researchers have been looking for environmentally friendly methods to control pests whose populations are increasing because of climate change’s effects. In this context, plant breeders exploit resistance gene donors to improve susceptible elite varieties and create new resistant cultivars. For example, rice breeders have developed resistant lines using conventional and molecular breeding techniques to tackle insect pests [14,15,16], diseases [15,17,18], drought [19,20], etc.

Plants mostly use antixenosis and antibiosis to deter or reduce insect assaults. Antixenosis is a way for plants to stop insects from settling on them, as insects are attracted to plants for food, mating, laying eggs, and shelter [21]. Therefore, antibiosis is known as the reaction brought out after the insect has colonized the plant [22] and affects insect growth, development, reproduction, and biological functions [23].

Olfaction is one of the primary information sources used by insects to identify and locate various resources such as hosts, predators, and partners [24]. Olfactory cues are signals that are reliable indicators of the behavior of insects [25]. Olfactory cues contribute to localization of host plants even in a complex environment, when the nervous system of the insect receives volatile information at fine-scale spatiotemporal resolution [26,27]. Host odors may be released in different ways, either through qualitative blends of volatile compounds [28,29] or quantitative mixtures [30,31]. Additionally, the relative variability of these compounds can help distinguish hosts from non-hosts [32]. Early studies showed that olfactometers may be highly effective in determining relative preference for various odor sources [33,34,35].

Olfactometers and wind tunnels are the tools mostly used to monitor the responses of insects to odor cues [36]. Multiple arm olfactometers, like Y-tube, U-tube, four-arm, and six-arm ones, are often used to detect and measure insect responses to odor cues. To our knowledge, no research study has explored the behavior of SEFs in the presence of rice genotypes. However, knowledge of these bio-ecological parameters will play an important role in the identification of resistant hosts and pest control in Africa.

This study investigated the response of SEFs to the cues emitted by leaves from four rice varieties (ITA306, WAB56-104, CG14, and RAM55) using a Y-arm olfactometer and screened cage techniques to elicit SEF host-seeking responses to rice. Finally, it will help (i) use rice genetic diversity to control SEF, (ii) understand the basis of SEF preferences, and (iii) discover resistant donors for breeding programs to develop new lines as an environmentally friendly solution against SEF.

## 2. Material and Methods

### 2.1. Field and Leaf Sampling

Rice plants were grown at the AfricaRice-Benin station (06°25.256 N, 002°19.765 E, and 15 m above sea level) under screened tents (3 m × 3 m) to avoid infestation by other insect species. The distance between two consecutive plants was 20 cm. Seeds were manually sown at a rate of three seeds per hill. Thinning to one plant per hill was performed 10 days after sowing (DAS). The application of 200 kg ha^−1^ NPK (14-14-14) was performed 14 DAS, and 50 kg ha^−1^ of urea was applied 5 weeks after sowing. Irrigation and weed control were performed when required.

Young leaves used in the experiments were sampled from 4- or 5-week-old rice plants of the four varieties in the screened cages. Twenty grams of fresh leaves were sampled from each variety for the Y-tube olfactometer study. Table 1 shows the varietal status from an early field assessment where these four rice accessions had been submitted to SEF attack.

### 2.2. Insects Used in the Study

Only healthy SEF female adults were used in the experiment. They were captured in rice fields at the AfricaRice-Benin station and immediately transferred to the laboratory, where they were submitted to the behavioral study. Hence, individual females were enclosed in tubes and kept in the laboratory. Each female was tested once to avoid any stress due to overmanipulating that could mislead its behavior.

### 2.3. Y-Tube Olfactometer Experiments

The attraction of SEFs to odor sources was studied in two-choice tests using a Y-tube olfactometer for 10 odor source combinations. For the first series of experiments, odors from the leaves of each rice variety were tested 60 times against clean air, as follows: ITA306 vs. clean air, WAB56-104 vs. clean air, CG14 vs. clean air, and RAM55 vs. clean air. For the second series, odors produced by the leaves of the different rice varieties were compared between 60 and 80 times against other varieties, including ITA306 vs. WAB56-104, CG14 vs. WAB56-104, RAM55 vs. WAB56-104, ITA306 vs. CG14, ITA306 vs. RAM55, and RAM55 vs. CG14.

The Y-tube olfactometer (SERBATOI AUTOCLAVI, Type ELTO, Vol. 50) system that created an airstream was like the one explained by Gnanvossou et al. [34] and Piesik et al. [37]. This Y-tube olfactometer setup has been extensively used for studying the olfactory responses of many insects [33,38].

Female SEFs were individually enclosed in plastic tubes (10 mm in diameter and 1000 mm long) and starved for 1 h at 25–28 °C temperature and 65–90% relative humidity (RH) prior to the olfactometer bioassays. For the behavioral study, each female was placed at the base of an iron wire positioned in the middle of the Y-shaped glass tube and parallel to the tube walls. The wind speed in the olfactometer arms was set at four liters per minute (4 L/min). Insect movement and orientation were observed until they reached the end of one of the arms, or for a maximum of 5 min, after which they were subsequently removed [34]. After each of the five tests, the positions of the odor sources were exchanged to correct any unforeseen asymmetry in the experimental setup. The number of females that had chosen or rebutted either odor source was recorded for each combination. A total of 60 to 80 females were tested on 3–4 consecutive days, with 20 females per day. New odor sources and new females were used for the daily tests.

Then, for each olfactometer test, we timed how long each insect took to make a decision. At the end of each evaluation, we summed together all of the recorded timings to calculate the total time spent on each choice during the comparison. We utilized a digital chronometer for this.

### 2.4. Preparation of Odor Sources Used for the Y-Tube Olfactometer

The leaves used as odor sources in the Y-tube olfactometer bioassays were cut from rice plants in the morning and immediately stored in a cooler containing ice packs prepared for the experiments. The cooler preserves the leaves’ freshness and keeps them hydrated during the experiments. For each assessment, 20 g of fresh leaves were used.

### 2.5. Screened Cage Free Choice Experiments

At the AfricaRice-Benin station, a rearing screen cage housed three potted rice plants for the experiment. The pots were plastic containers with a 2 L capacity. They were filled with soil (two-thirds topsoil plus one-third compost) and kept in an insect-proof screen cage. Several plants were planted at a density of six seeds per pot for each rice variety. After 21 days, thinning was performed to yield three plants per pot, followed by the application of 2 g of NPK 45-45-45. The host selection test started 40 days after sowing. Therefore, two pots from two different rice varieties were placed on opposite sides of the screened cage before releasing the SEF female in the middle of it. The movement and orientation of the insects were assessed for 5 min from the release of the insect to the choice of the host plant. When the insect reached its host and remained on it for 5 min, the choice was confirmed. If the female did not reach its host plant after 5 min, this choice was not validated and was therefore a “no-choice” event. The number of observations completed was 80 for CG14 vs. RAM55 and CG14 vs. WAB56-104 and 100 for ITA306 vs. WAB56-104 vs. CG14 vs. RAM55. After five observations, the pot position was changed to avoid a biased conclusion. The procedure followed that of Zakir et al. [39], who studied the resistance of cotton plants against *Spodoptera littoralis*.

As for the olfactometer test, we determined the time taken by SEFs to make a decision.

### 2.6. Statistical Analyses

Data analysis was performed with the open-source statistical software R version 4.5.0 (R Core Team, 2025, Vienna, Austria) [40]. The number of SEFs that chose each odor source was analyzed using binomial tests with the *binom.test* function, with the null hypothesis of equal distribution across Y-tube olfactometer arms. Differences between SEF groups were tested via 2 × 2 contingency table analysis using the *chisq.test* function. Non-responding females were excluded from the analysis.

The time spent in each Y-tube arm was compared using analysis of variance (ANOVA) implemented with the *aov* function. For the potted plant experiments, data were processed identically to the Y-tube trials. All analyses utilized functions from the stats package (R Core Team, 2025) [40] within the R environment.

## 3. Results

### 3.1. Behavior of SEFs in Response to Rice Variety Leaf Cues Paired with Clean Air

**Figure 1:**
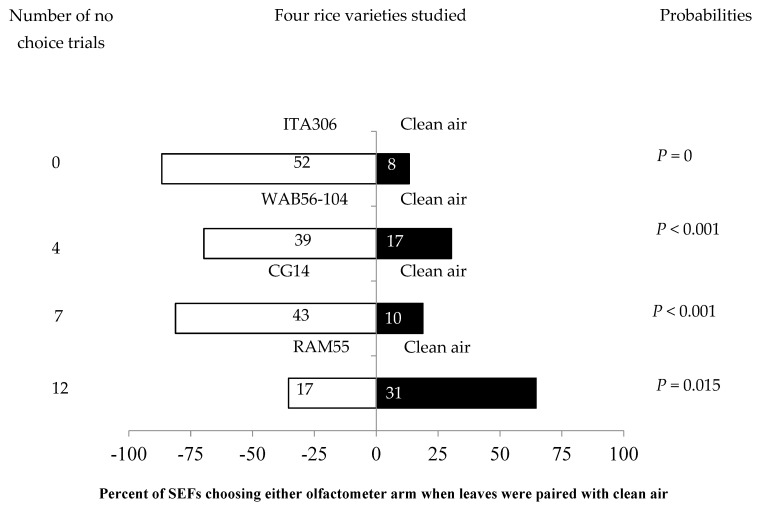
Response of SEFs when offered choices between different rice varieties and clean air in a Y-tube olfactometer. The numbers in the bars represent the total number of females that chose either olfactometer arm. The probabilities given to the right of the bars are for the binomial test.

The four rice varieties showed different levels of attractiveness to the SEFs when paired with clean air. Figure 1 shows the number of attracted females. SEFs were significantly attracted to the leaf odor cues of each of three rice varieties (ITA306, WAB56-104, and CG14) when they were paired with clean air. Therefore, rice accession RAM 55 repelled SEF massively during the test. Also, the number of “no choice” records varied according to the variety tested (Figure 1).

### 3.2. SEF Response to Rice Leaves from Two Varieties Paired with One Another

**Figure 2:**
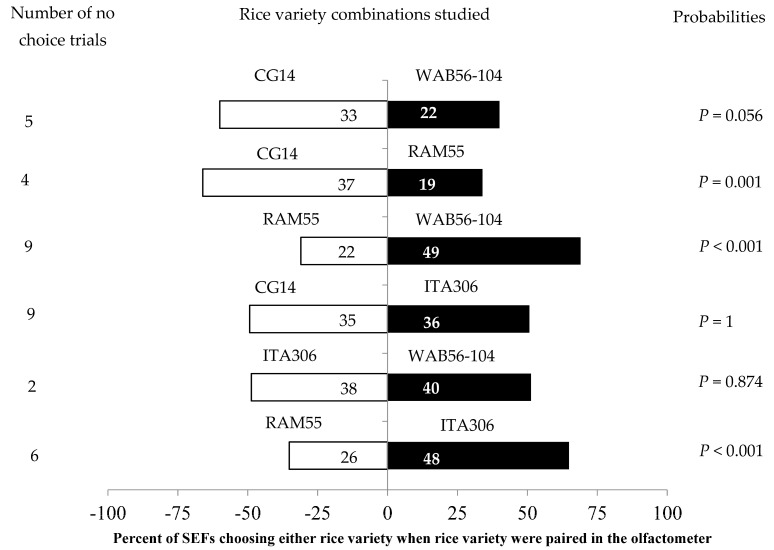
Response of SEFs when offered the choice between leaves from two rice varieties in a Y-tube olfactometer. The numbers in the bars represent the total number of females that chose either olfactometer arm. The probabilities given to the right of the bars are for the binomial test.

The leaves of two given varieties differently attracted SEF females when paired with each other (Figure 2). SEFs showed significant preferences in the following tests: RAM55 vs. ITA306; CG14 vs. RAM55; and RAM55 vs. WAB56-104 (Figure 2). The African rice varieties CG14 and WAB56-104 significantly attracted the insects during this test, while accession RAM55 repelled them. No significant differences were observed between ITA306 vs. WAB56-104, CG14 vs. WAB56-104, or CG14 vs. ITA306. Also, the number of “no choice” trials varied according to the variety tested (Figure 2).

### 3.3. SEF Time Spent Before Reaching the Hosts in the Y-Tube Olfactometer

Table 2 shows that there was a significant difference in the time spent by SEFs on the choice between CG14 vs. clean air. SEFs spent less time reaching the CG14 leaves. There was no major difference in the time during ITA306 vs. clean air, WAB56-104 vs. clean air, and RAM 55 vs. clean air assessments. Table 2 does not exhibit any difference in the time spent by SEFs to reach each accession when paired with another accession.

### 3.4. SEF Response to Potted Rice Plants When Two Varieties Were Paired in Cage

**Figure 3:**
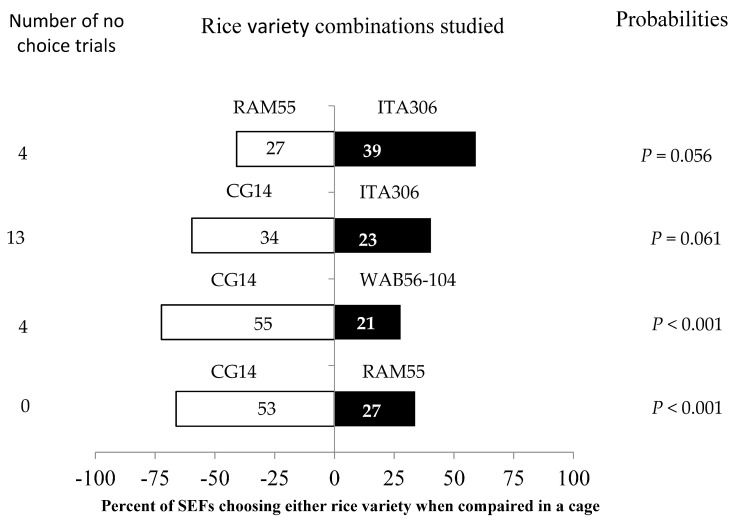
Response of SEFs when offered the choice between potted plants from two rice varieties in a screened cage. The numbers in the bars represent the total number of females that chose either rice variety. The probabilities given to the right of the bars are for the binomial test.

SEFs displayed different levels of preference for each of the tested rice varieties in the screened cage experiment when paired with each other (Figure 3). In this evaluation, the African rice variety CG14 attracted more female insects when paired with WAB56-104 and RAM55. However, RAM55 and CG14 showed no significant difference when paired with ITA306. Furthermore, the number of “no choice” trials varied according to the tested varieties. 

### 3.5. SEF Response to Potted Rice Plants When Four Varieties Were Paired in Cage

**Figure 4:**
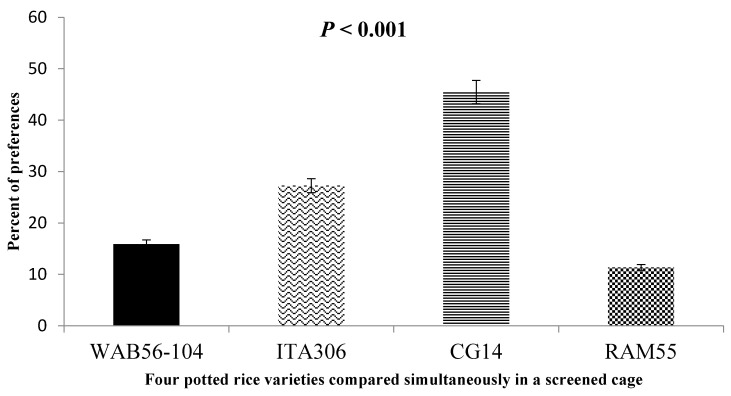
Response of SEFs when offered the choice among potted plants from four rice varieties in a screened cage.

When given a choice inside the screened cage, Figure 4 illustrates the attraction of SEF females to the four rice varieties tested. The results demonstrated a clear difference in insect preference for some genotypes compared to others, as follows: RAM 55 < WAB56-104 < ITA306 < CG14.

### 3.6. SEF Time Spent Before Reaching the Host Plants in the Screen Cage

There was no significant difference in the time spent by females before reaching the host plants when paired with each other in the screen cage.

## 4. Discussion

This study’s findings show the importance of rice leaf cues in the host selection process by SEFs when each rice variety was paired with clean air in the olfactometer. Female SEFs were able to easily choose leaf cues emitted by the rice varieties CG14, WAB56-104, and ITA306 when paired with clean air. In the test of rice variety RAM55 paired with clean air, SEF significantly avoided the leaf cues from the rice variety. Therefore, varieties CG14, WAB56-104, and ITA306 could be considered the most attractive to SSF, while variety RAM55 showed a repellent action to SEFs. This result seems to demonstrate that varieties WAB56-104 and ITA306 are more susceptible to attack by SEF. This outcome corroborated the finding of Togola et al. [7], who reported that WAB56-104 was highly susceptible to SEF. When leaves of the ITA306 rice variety were paired with those of WAB56-104, no significant difference was found, suggesting similarity between these two rice varieties. Therefore, WAB56-104 and ITA306 can be considered susceptible to SEFs. ITA306 is also known as being susceptible to the African rice gall midge (AfRGM), which also belongs to the Diptera order, like SEF [41].

SEFs were attracted to leaves of the CG14 rice variety when tested against clean air. Also, when paired with other rice varieties, CG14 was preferred by the insects. This confirms the findings of Bocco et al. [42], revealing a high content of flavonoids in CG14. This allelochemical modulates the feeding and oviposition behavior of SEF insects. Once these insects choose this variety to stay and lay their eggs, its high contents of tannins, saponosides, and reducing sugars acts as poisons by blocking egg development [42]. These results suggest that CG14 can be considered as a trap variety by attracting SEF females to lay their eggs; however, some early studies under field and semi-controlled conditions identified the variety as resistant to SEFs [7,8,42]. This finding suggests that CG14 could be considered as a trap variety because it attracts SEF females to lay their eggs. We can assume that larvae did not survive after hatching on CG14, as evidenced by our screening under a tent [8], suggesting a potential antibiosis effect.

The behavior of SEFs in the olfactometer showed a significant difference in response to RAM55 versus clean air. Volatile substances that repel SEFs during the search for host plants could influence the insect’s choice preference. This finding can be considered antixenosis, which is known as resistance exerted by plants that deters or reduces colonization by insects searching the plant for food, mating, ovipositioning, or shelter [43]. This resistance can be biophysical, biochemical, or a combination of both, resulting in a reduction of the initial number of insects in subsequent generations. Antixenosis is complex and combines morphological, physical, and structural qualities, which include pubescence and tissue hardness, which limit insect mobility [44], repellents and anti-feedings [45], and plant stimuli [46]. Past research by Bocco et al. [42] revealed the presence of terpenes in RAM55. According to Laothawornkitkul et al. [47], Unsicker et al. [48], and Maffei [49], some terpenoids serve as repellents and protect plants against herbivore arthropods.

When the rice varieties were paired with each other in the Y-tube olfactometer, several behaviors were noticed in insects. No difference was found between ITA306, WAB56-104, and CG14 in terms of SEF preference. Therefore, SEFs avoided RAM55 and were attracted by ITA306, WAB56-104, and CG14. These differences in the behavior of SEFs when offered rice leaf volatiles could be explained by the fact that these rice varieties have in common a few compounds that confer an attraction or repellence effect. Various allelochemical substances, which play a crucial protective role against insects, are unrelated to pest resistance [50,51,52]. The same conclusion was reached in a study on the total polyphenol, total nitrogen, soluble nitrogen, and soluble sugar contents of 29 Asian rice gall midge (AsRGM)-resistant and -susceptible rice varieties [53].

The screened cage experiments revealed that ITA306, WAB56-104, and CG14 plants attracted SEFs. Assessment of CG14, WAB56-104, CG14, and RAM55 in a cage confirmed the previously stated attractiveness of these three varieties. The last experiment in a cage elucidated the interactions between each of them and SEFs. African rice variety RAM55 repels SEFs, which avoid laying their eggs on it to preserve their progeny, while CG14 attracts SEF females to lay eggs on it and acts as a lure, poisoning larvae after hatching. SEF adult preference for a plant might be based on olfactory stimuli, which also depend on the metabolites and chemical compounds produced by the host plant. Generally, female insects prefer to lay their eggs on plants where their young can grow easily [54] or on plants that provide better nutrients for their young’s growth [55,56,57]. Most researchers prefer using varieties with saponin content, as this component has repellent or intoxicant effects on insects (Morrissey and Osbourn [58], cited by Francis et al. [59]). Females often search for adequate food for their offspring’s development. Such behavior may support the preference of females for ITA306 and WAB56-104 leaves instead of RAM55 and CG14. The resistance of CG14 mentioned by Togola et al. [60] and Bocco et al. [42] after screening must be considered, because of its inability to provide survival to insect offspring. The current research did not analyze the chemical composition of CG14, but Bocco et al. [42] reported the presence of heterosides and saponosides in this African rice accession. The resistance found in plants is very complex, because it also depends on the environment. Coevolution between African rice varieties (CG14, RAM55) and the surrounding biota––including viruses, fungi, bacteria, nematodes, insects, and mammals––could certainly have generated adaptation without influences on the population’s mean fitness [61,62]. In general, antibiosis reactions are related to the presence and activity of endophytic microorganisms that produce repulsive or toxic compounds [63,64]. Studying endophytes in African rice tissues would show a difference between plants that are vulnerable and those that are resistant [65]. As a result of this difference, bioassays would determine the behavior of the insect against each microorganism using an olfactometer. The two resistant rice accessions, CG14 and RAM55, used for this study confirm that this species is a reservoir of resistance genes, as mentioned by early research [66,67,68].

This study used a Y-arm olfactometer and a screened cage to examine how SEF responds to chemicals released by leaves from four types of rice (ITA306, WAB56-104, CG14, and RAM55). Breeding tools can employ genetic diversity to provide a long-term alternative for SEF management. Most of the data acquired during the study on SEFs’ time spent seeking cue sources showed no consistent changes. The results of the SEF behavioral investigation through a Y-tube olfactometer and a screened cage supported the varieties’ status during early field screening. As a result, the findings of the behavioral assessments conducted using both the olfactometer and cage were very similar. The screened cage assessment also provided a superior evaluation of SEF behavior and was simpler to carry out than the olfactometer assessment. The cage observations demonstrated that visual signals played a crucial role in SEF host preference. If RAM55’s repellent property is proven by further research, it might be planted between susceptible genotypes to protect them against SEF attacks. Rice cultivars CG14 and RAM55 have the potential to contribute to a breeding program to boost rice resistance to SEFs.

## 5. Conclusions

The signals from CG14, WAB56-104, and ITA306 strongly attracted female SEFs in olfactometer and cage tests, while RAM55 repulsed the insects. The attractiveness of WAB56-104 and ITA306 confirmed their susceptibility, as mentioned in earlier studies. Therefore, African rice CG14, which draws in SEFs, should be considered as bait, because it strongly attracts SEF females to lay eggs and also prevents the larvae from growing. Farmers can plant CG14 as bait to preserve susceptible varieties while using RAM55 to safeguard rice varieties that are vulnerable, because it exhibits repellence. The olfactometer and cage tests offered a thorough description of the status of rice accessions, revealed following multiple earlier screen cage evaluations. They helped us better grasp the processes governing plant insect resistance. The aforementioned tools cannot replace the field and screen house techniques originally utilized by plant breeders to detect resistant donors without providing any information about the source of resistance. CG14 and RAM55 are potential candidates for breeding programs to develop new lines that are resistant to SEF attacks.

## Figures and Tables

**Table 1:** Characteristics of the four rice varieties used for odor-mediated studies.

Varieties	Species	Ecologies	Reaction to SEF
ITA306	*Oryza sativa*	Lowland/Irrigated	Susceptible
WAB56-104	*Oryza sativa*	Upland	Susceptible
CG14	*Oryza glaberrima*	Floating	Resistant
RAM55	*Oryza glaberrima*	Floating	Resistant

**Table 2:** Mean staying time (second) of SEF with each of the cues during Y-tube olfactometer bioassays with rice varieties and their *p*-values.

ITA306	WAB56-104	CG14	RAM55	Clean Air	*p*-Values
		39.98		66.30	0.007
38.65				65.25	0.264
			28.65	42.48	0.365
	39.71			45.15	0.773
84.33		90.03			0.636
		59	66.16		0.393
36.30			33.38		0.787
	73	75.33			0.752
36.97	45.85				0.474
	57.82		56.02		0.915

## Data Availability

The original contributions presented in this study are included in the article/Supplementary Materials. Further inquiries can be directed to the corresponding author.

## Supplementary Materials

The following supporting information can be downloaded at: https://www.mdpi.com/article/10.3390/insects16080752/s1, Table S1. ITA306 vs. Clean air assessment; Table S2. WAB56-104 vs. Clean air assessment; Table S3. CG14 vs. Clean air assessment; Table S4. CG14 vs. Clean air assessment; Table S5. WAB56-104 vs. ITA306 assessment; Table S6. RAM55 vs. ITA306 assessment; Table S7. CG14 vs. ITA306 assessment; Table S8. WAB56-104 vs. RAM55 assessment; Table S9. CG14 vs. WAB56-104 assessment; Table S10. CG14 vs. RAM55 assessment; Table S11. Mean staying time.
