# Supplementary material for: Determination of Rice Accession Status Using Infochemical and Visual Cues Emitted to Sustainably Control Diopsis apicalis Dalman
Source: Insects. 2025 Jul 23;16(8):752. doi: 10.3390/insects16080752 (PMC12386945; doi:10.3390/insects16080752)
Supplement: Supplementary file 1 [file insects-16-00752-s001.zip › Table S1. ITA306 vs Clean air assessment.pdf]

Test N°	ITA 306	ITA arm duration	Clean air
1	1	15	0
2	1	21	0
3	0		1
4	1	82	0
5	1	126	0
6	1	3	0
7	1	125	0
8	1	53	0
9	1	228	0
10	1	3	0
11	1	2	0
12	1	2	0
13	1	3	0
14	1	194	0
15	0		1
16	1	56	0
17	1	88	0
18	1	1	0
19	1	14	0
20	1	3	0
21	0		1
22	0		1
23	1	136	0
24	1	2	0
25	1	159	0
26	1	5	0
27	0		1
28	1	3	0
29	1	22	0
30	1	6	0
31	1	5	0
32	1	5	0
33	1	3	0
34	1	6	0
35	1	2	0
36	1	17	0
37	1	4	0
38	1	11	0
39	1	3	0
40	1	12	0
41	1	4	0
42	1	5	0
43	1	123	0
44	1	2	0
45	1	64	0
46	1	5	0

47	1	7	0
48	1	149	0
49	1	61	0
50	1	18	0
51	1	8	0
52	1	73	0
53	1	3	0
54	1	9	0
55	0		1
56	1	13	0
57	1	7	0
58	0		1
59	1	87	0
60	0		1
**Total**	**52**		**8**
Mean duration	63.63	**39.58**	65.25
Percentage	87		13
Speed	2.04		1.99
Standard dev	0.547722558	44.5570795	0.547722558

Air Column Conv.	No choice	Speed ITA arm duration (mm/s)
	0	1.23895239
	0	0.619476195
154	0	
	0	1.585365854
	0	1.317463175
	0	4.333333333
	0	1.4
	0	2.452831887
	0	0.571754386
	0	0.433333333
	0	0.65
	0	6.5
	0	0.433333333
	0	0.671392784
37	0	
	0	2.321428571
	0	1.477272727
	0	13
	0	9.285714286
	0	43.33333333
48	0	
18	0	
	0	0.955882353
	0	65
	0	0.817616289
	0	26
7	0	
	0	4.333333333
	0	5.99999991
	0	21.66666667
	0	26
	0	26
	0	4.333333333
	0	21.66666667
	0	65
	0	7.647588235
	0	32.5
	0	11.81818182
	0	43.33333333
	0	1.833333333
	0	32.5
	0	2.6
	0	1.569156916
	0	65
	0	2.3125
	0	26

	0	18.57142857
	0	0.872483221
	0	2.13114755
	0	7.222222222
	0	16.25
	0	1.788219179
	0	4.333333333
	0	14.44444444
4	0	
	0	1
	0	18.57142857
252	0	
	0	1.494252874
2	0	
	**0**	
**65.25**		
176.7766953	0	10.00524294

Speed Air Column (mm/s)

0.844155844

3.513513514

2.783333333

7.222222222

18.57142857

32.5

0.515873159

65

45.59716337
