# Supplementary material for: Determination of Rice Accession Status Using Infochemical and Visual Cues Emitted to Sustainably Control Diopsis apicalis Dalman
Source: Insects. 2025 Jul 23;16(8):752. doi: 10.3390/insects16080752 (PMC12386945; doi:10.3390/insects16080752)
Supplement: Supplementary file 1 [file insects-16-00752-s001.zip › Table S10. CG14 vs RAM55 assessment.pdf]

| N° | CG14 | Duration CG14 | RAM55 | Duration RAM55 | No Choice |
|---|---|---|---|---|---|
| 1 | 1 | 81 | | | |
| 2 | 1 | 37 | | | |
| 3 | 1 | 101 | | | |
| 4 | 1 | 60 | | | |
| 5 | 1 | 40 | | | |
| 6 | 1 | 71 | | | |
| 7 | | | 1 | 114 | |
| 8 | 1 | 25 | | | |
| 9 | | | 1 | 33 | |
| 10 | | | 1 | 45 | |
| 11 | 1 | 118 | | | |
| 12 | | | 1 | 55 | |
| 13 | 1 | 67 | | | |
| 14 | | | | | 1 |
| 15 | | | 1 | 82 | |
| 16 | 1 | 65 | | | |
| 17 | | | 1 | 53 | |
| 18 | 1 | 23 | | | |
| 19 | 1 | 70 | | | |
| 20 | 1 | 76 | | | |
| 21 | | | 1 | 54 | |
| 22 | | | 1 | 27 | |
| 23 | 1 | 55 | | | |
| 24 | 1 | 26 | | | |
| 25 | 1 | 37 | | | |
| 26 | | | 1 | 39 | |
| 27 | | | | | 1 |
| 28 | | | 1 | 59 | |
| 29 | 1 | 29 | | | |
| 30 | 1 | 50 | | | |
| 31 | 1 | 29 | | | |
| 32 | 1 | 50 | | | |
| 33 | 1 | 69 | | | |
| 34 | 1 | 47 | | | |
| 35 | | | 1 | 24 | |
| 36 | | | | | 1 |
| 37 | 1 | 42 | | | |
| 38 | 1 | 29 | | | |
| 39 | | | 1 | 46 | |
| 40 | 1 | 56 | | | |
| 41 | 1 | 34 | | | |
| 42 | | | 1 | 80 | |
| 43 | | | 1 | 128 | |
| 44 | 1 | 29 | | | |
| 45 | | | 1 | 29 | |
| 46 | 1 | 69 | | | |

| | | | | | | |
|---|---|---|---|---|---|---|
| | 47 | 1 | 99 | | | |
| | 48 | 1 | 119 | | | |
| | 49 | | | 1 | 70 | |
| | 50 | | | 1 | 141 | |
| | 51 | 1 | 58 | | | |
| | 52 | 1 | 56 | | | |
| | 53 | | | | | 1 |
| | 54 | | | 1 | 85 | |
| | 55 | 1 | 71 | | | |
| | 56 | 1 | 67 | | | |
| | 57 | 1 | 50 | | | |
| | 58 | 1 | 57 | | | |
| | 59 | | | 1 | 93 | |
| | 60 | 1 | 121 | | | |
| Percentage | | 66.07143 | | 33.9285714 | | |
| Tot choices/ Duration | | 37 | 59 | 19 | 66.15789474 | 4 |
