# Supplementary material for: Determination of Rice Accession Status Using Infochemical and Visual Cues Emitted to Sustainably Control Diopsis apicalis Dalman
Source: Insects. 2025 Jul 23;16(8):752. doi: 10.3390/insects16080752 (PMC12386945; doi:10.3390/insects16080752)
Supplement: Supplementary file 1 [file insects-16-00752-s001.zip › Table S11. Mean staying time.pdf]

Environment	Factor	Preferences	Times
CG14 vs Clean air	CG14	1	59
CG14 vs Clean air	CG14	1	82
CG14 vs Clean air	CG14	1	45
CG14 vs Clean air	CG14	1	58
CG14 vs Clean air	CG14	1	35
CG14 vs Clean air	CG14	1	44
CG14 vs Clean air	CG14	1	62
CG14 vs Clean air	CG14	1	36
CG14 vs Clean air	CG14	1	27
CG14 vs Clean air	CG14	1	39
CG14 vs Clean air	CG14	1	46
CG14 vs Clean air	CG14	1	60
CG14 vs Clean air	CG14	1	45
CG14 vs Clean air	CG14	1	47
CG14 vs Clean air	CG14	1	30
CG14 vs Clean air	CG14	1	29
CG14 vs Clean air	CG14	1	21
CG14 vs Clean air	CG14	1	29
CG14 vs Clean air	CG14	1	19
CG14 vs Clean air	CG14	1	32
CG14 vs Clean air	CG14	1	22
CG14 vs Clean air	CG14	1	20
CG14 vs Clean air	CG14	1	15
CG14 vs Clean air	CG14	1	17
CG14 vs Clean air	CG14	1	19
CG14 vs Clean air	CG14	1	27
CG14 vs Clean air	CG14	1	29
CG14 vs Clean air	CG14	1	19
CG14 vs Clean air	CG14	1	35
CG14 vs Clean air	CG14	1	18
CG14 vs Clean air	CG14	1	27
CG14 vs Clean air	CG14	1	20
CG14 vs Clean air	CG14	1	57
CG14 vs Clean air	CG14	1	75
CG14 vs Clean air	CG14	1	85
CG14 vs Clean air	CG14	1	24
CG14 vs Clean air	CG14	1	18
CG14 vs Clean air	CG14	1	91
CG14 vs Clean air	CG14	1	98
CG14 vs Clean air	CG14	1	20
CG14 vs Clean air	CG14	1	67
CG14 vs Clean air	CG14	1	26
CG14 vs Clean air	CG14	1	45
CG14 vs Clean air	Clean Air	1	63
CG14 vs Clean air	Clean Air	1	82
CG14 vs Clean air	Clean Air	1	63

CG14 vs Clean air	Clean Air	1	27
CG14 vs Clean air	Clean Air	1	29
CG14 vs Clean air	Clean Air	1	137
CG14 vs Clean air	Clean Air	1	20
CG14 vs Clean air	Clean Air	1	33
CG14 vs Clean air	Clean Air	1	133
CG14 vs Clean air	Clean Air	1	76
ITA306 vs Clean air	ITA306	1	15
ITA306 vs Clean air	ITA306	1	21
ITA306 vs Clean air	ITA306	1	82
ITA306 vs Clean air	ITA306	1	126
ITA306 vs Clean air	ITA306	1	3
ITA306 vs Clean air	ITA306	1	125
ITA306 vs Clean air	ITA306	1	53
ITA306 vs Clean air	ITA306	1	228
ITA306 vs Clean air	ITA306	1	3
ITA306 vs Clean air	ITA306	1	2
ITA306 vs Clean air	ITA306	1	2
ITA306 vs Clean air	ITA306	1	3
ITA306 vs Clean air	ITA306	1	194
ITA306 vs Clean air	ITA306	1	56
ITA306 vs Clean air	ITA306	1	88
ITA306 vs Clean air	ITA306	1	1
ITA306 vs Clean air	ITA306	1	14
ITA306 vs Clean air	ITA306	1	3
ITA306 vs Clean air	ITA306	1	136
ITA306 vs Clean air	ITA306	1	2
ITA306 vs Clean air	ITA306	1	159
ITA306 vs Clean air	ITA306	1	5
ITA306 vs Clean air	ITA306	1	3
ITA306 vs Clean air	ITA306	1	22
ITA306 vs Clean air	ITA306	1	6
ITA306 vs Clean air	ITA306	1	5
ITA306 vs Clean air	ITA306	1	5
ITA306 vs Clean air	ITA306	1	3
ITA306 vs Clean air	ITA306	1	6
ITA306 vs Clean air	ITA306	1	2
ITA306 vs Clean air	ITA306	1	17
ITA306 vs Clean air	ITA306	1	4
ITA306 vs Clean air	ITA306	1	11
ITA306 vs Clean air	ITA306	1	3
ITA306 vs Clean air	ITA306	1	12
ITA306 vs Clean air	ITA306	1	4
ITA306 vs Clean air	ITA306	1	5
ITA306 vs Clean air	ITA306	1	123
ITA306 vs Clean air	ITA306	1	2
ITA306 vs Clean air	ITA306	1	64

ITA306 vs Clean air	ITA306	1	5
ITA306 vs Clean air	ITA306	1	7
ITA306 vs Clean air	ITA306	1	149
ITA306 vs Clean air	ITA306	1	61
ITA306 vs Clean air	ITA306	1	18
ITA306 vs Clean air	ITA306	1	8
ITA306 vs Clean air	ITA306	1	73
ITA306 vs Clean air	ITA306	1	3
ITA306 vs Clean air	ITA306	1	9
ITA306 vs Clean air	ITA306	1	13
ITA306 vs Clean air	ITA306	1	7
ITA306 vs Clean air	Clean air	1	154
ITA306 vs Clean air	Clean air	1	37
ITA306 vs Clean air	Clean air	1	48
ITA306 vs Clean air	Clean air	1	18
ITA306 vs Clean air	Clean air	1	7
ITA306 vs Clean air	Clean air	1	4
ITA306 vs Clean air	Clean air	1	252
ITA306 vs Clean air	Clean air	1	2
RAM55 vs Clean air	RAM55	1	12
RAM55 vs Clean air	RAM55	1	2
RAM55 vs Clean air	RAM55	1	9
RAM55 vs Clean air	RAM55	1	6
RAM55 vs Clean air	RAM55	1	13
RAM55 vs Clean air	RAM55	1	21
RAM55 vs Clean air	RAM55	1	23
RAM55 vs Clean air	RAM55	1	7
RAM55 vs Clean air	RAM55	1	55
RAM55 vs Clean air	RAM55	1	53
RAM55 vs Clean air	RAM55	1	167
RAM55 vs Clean air	RAM55	1	9
RAM55 vs Clean air	RAM55	1	16
RAM55 vs Clean air	RAM55	1	3
RAM55 vs Clean air	RAM55	1	7
RAM55 vs Clean air	RAM55	1	55
RAM55 vs Clean air	RAM55	1	29
RAM55 vs Clean air	Clean air	1	6
RAM55 vs Clean air	Clean air	1	23
RAM55 vs Clean air	Clean air	1	237
RAM55 vs Clean air	Clean air	1	11
RAM55 vs Clean air	Clean air	1	26
RAM55 vs Clean air	Clean air	1	4
RAM55 vs Clean air	Clean air	1	75
RAM55 vs Clean air	Clean air	1	89
RAM55 vs Clean air	Clean air	1	64
RAM55 vs Clean air	Clean air	1	11
RAM55 vs Clean air	Clean air	1	53

RAM55 vs Clean air	Clean air	1	4
RAM55 vs Clean air	Clean air	1	195
RAM55 vs Clean air	Clean air	1	6
RAM55 vs Clean air	Clean air	1	35
RAM55 vs Clean air	Clean air	1	28
RAM55 vs Clean air	Clean air	1	75
RAM55 vs Clean air	Clean air	1	21
RAM55 vs Clean air	Clean air	1	67
RAM55 vs Clean air	Clean air	1	4
RAM55 vs Clean air	Clean air	1	21
RAM55 vs Clean air	Clean air	1	26
RAM55 vs Clean air	Clean air	1	4
RAM55 vs Clean air	Clean air	1	34
RAM55 vs Clean air	Clean air	1	13
RAM55 vs Clean air	Clean air	1	21
RAM55 vs Clean air	Clean air	1	2
RAM55 vs Clean air	Clean air	1	49
RAM55 vs Clean air	Clean air	1	25
RAM55 vs Clean air	Clean air	1	9
RAM55 vs Clean air	Clean air	1	79
WAB56-104 vs Clean air	WAB56-104	1	26
WAB56-104 vs Clean air	WAB56-104	1	22
WAB56-104 vs Clean air	WAB56-104	1	3
WAB56-104 vs Clean air	WAB56-104	1	2
WAB56-104 vs Clean air	WAB56-104	1	5
WAB56-104 vs Clean air	WAB56-104	1	27
WAB56-104 vs Clean air	WAB56-104	1	82
WAB56-104 vs Clean air	WAB56-104	1	8
WAB56-104 vs Clean air	WAB56-104	1	147
WAB56-104 vs Clean air	WAB56-104	1	95
WAB56-104 vs Clean air	WAB56-104	1	6
WAB56-104 vs Clean air	WAB56-104	1	18
WAB56-104 vs Clean air	WAB56-104	1	4
WAB56-104 vs Clean air	WAB56-104	1	13
WAB56-104 vs Clean air	WAB56-104	1	12
WAB56-104 vs Clean air	WAB56-104	1	6
WAB56-104 vs Clean air	WAB56-104	1	3
WAB56-104 vs Clean air	WAB56-104	1	165
WAB56-104 vs Clean air	WAB56-104	1	9
WAB56-104 vs Clean air	WAB56-104	1	42
WAB56-104 vs Clean air	WAB56-104	1	5
WAB56-104 vs Clean air	WAB56-104	1	1
WAB56-104 vs Clean air	WAB56-104	1	2
WAB56-104 vs Clean air	WAB56-104	1	5
WAB56-104 vs Clean air	WAB56-104	1	12
WAB56-104 vs Clean air	WAB56-104	1	37
WAB56-104 vs Clean air	WAB56-104	1	2

WAB56-104 vs Clean air	WAB56-104	1	8
WAB56-104 vs Clean air	WAB56-104	1	99
WAB56-104 vs Clean air	WAB56-104	1	6
WAB56-104 vs Clean air	WAB56-104	1	51
WAB56-104 vs Clean air	WAB56-104	1	119
WAB56-104 vs Clean air	WAB56-104	1	3
WAB56-104 vs Clean air	WAB56-104	1	132
WAB56-104 vs Clean air	WAB56-104	1	191
WAB56-104 vs Clean air	WAB56-104	1	145
WAB56-104 vs Clean air	WAB56-104	1	227
WAB56-104 vs Clean air	WAB56-104	1	6
WAB56-104 vs Clean air	WAB56-104	1	15
WAB56-104 vs Clean air	Clean air	1	16
WAB56-104 vs Clean air	Clean air	1	138
WAB56-104 vs Clean air	Clean air	1	6
WAB56-104 vs Clean air	Clean air	1	5
WAB56-104 vs Clean air	Clean air	1	5
WAB56-104 vs Clean air	Clean air	1	13
WAB56-104 vs Clean air	Clean air	1	4
WAB56-104 vs Clean air	Clean air	1	2
WAB56-104 vs Clean air	Clean air	1	32
WAB56-104 vs Clean air	Clean air	1	13
WAB56-104 vs Clean air	Clean air	1	277
WAB56-104 vs Clean air	Clean air	1	6
WAB56-104 vs Clean air	Clean air	1	17
WAB56-104 vs Clean air	Clean air	1	3
WAB56-104 vs Clean air	Clean air	1	86
WAB56-104 vs Clean air	Clean air	1	11
WAB56-104 vs Clean air	Clean air	1	41
TOG5681 vs Clean air	TOG5681	1	150
TOG5681 vs Clean air	TOG5681	1	80
TOG5681 vs Clean air	TOG5681	1	121
TOG5681 vs Clean air	TOG5681	1	63
TOG5681 vs Clean air	TOG5681	1	57
TOG5681 vs Clean air	TOG5681	1	211
TOG5681 vs Clean air	TOG5681	1	49
TOG5681 vs Clean air	TOG5681	1	76
TOG5681 vs Clean air	TOG5681	1	110
TOG5681 vs Clean air	TOG5681	1	140
TOG5681 vs Clean air	TOG5681	1	49
TOG5681 vs Clean air	TOG5681	1	67
TOG5681 vs Clean air	TOG5681	1	201
TOG5681 vs Clean air	TOG5681	1	215
TOG5681 vs Clean air	TOG5681	1	130
TOG5681 vs Clean air	TOG5681	1	125
TOG5681 vs Clean air	TOG5681	1	80
TOG5681 vs Clean air	TOG5681	1	135

TOG5681 vs Clean air	TOG5681	1	230
TOG5681 vs Clean air	TOG5681	1	118
TOG5681 vs Clean air	TOG5681	1	149
TOG5681 vs Clean air	TOG5681	1	53
TOG5681 vs Clean air	TOG5681	1	145
TOG5681 vs Clean air	TOG5681	1	139
TOG5681 vs Clean air	TOG5681	1	88
TOG5681 vs Clean air	TOG5681	1	103
TOG5681 vs Clean air	TOG5681	1	109
TOG5681 vs Clean air	TOG5681	1	81
TOG5681 vs Clean air	TOG5681	1	127
TOG5681 vs Clean air	TOG5681	1	94
TOG5681 vs Clean air	TOG5681	1	169
TOG5681 vs Clean air	TOG5681	1	90
TOG5681 vs Clean air	Clean air	1	178
TOG5681 vs Clean air	Clean air	1	184
TOG5681 vs Clean air	Clean air	1	36
TOG5681 vs Clean air	Clean air	1	72
TOG5681 vs Clean air	Clean air	1	125
TOG5681 vs Clean air	Clean air	1	100
TOG5681 vs Clean air	Clean air	1	130
TOG5681 vs Clean air	Clean air	1	138
TOG5681 vs Clean air	Clean air	1	102
TOG5681 vs Clean air	Clean air	1	133
TOG5681 vs Clean air	Clean air	1	211
TOG5681 vs Clean air	Clean air	1	117
TOG5681 vs Clean air	Clean air	1	110
TOG5681 vs Clean air	Clean air	1	121
TOG5681 vs Clean air	Clean air	1	82
TOG5681 vs Clean air	Clean air	1	96
TOG5681 vs Clean air	Clean air	1	105
TOG5681 vs Clean air	Clean air	1	131
TOG5681 vs Clean air	Clean air	1	181
TOG5681 vs Clean air	Clean air	1	91
TOG5681 vs Clean air	Clean air	1	115
TOG5681 vs Clean air	Clean air	1	166
RAM55 vs ITA306	RAM55	1	4
RAM55 vs ITA306	RAM55	1	43
RAM55 vs ITA306	RAM55	1	41
RAM55 vs ITA306	RAM55	1	82
RAM55 vs ITA306	RAM55	1	12
RAM55 vs ITA306	RAM55	1	43
RAM55 vs ITA306	RAM55	1	3
RAM55 vs ITA306	RAM55	1	41
RAM55 vs ITA306	RAM55	1	1
RAM55 vs ITA306	RAM55	1	19
RAM55 vs ITA306	RAM55	1	1

RAM55 vs ITA306	RAM55	1	12
RAM55 vs ITA306	RAM55	1	7
RAM55 vs ITA306	RAM55	1	138
RAM55 vs ITA306	RAM55	1	148
RAM55 vs ITA306	RAM55	1	5
RAM55 vs ITA306	RAM55	1	18
RAM55 vs ITA306	RAM55	1	22
RAM55 vs ITA306	RAM55	1	49
RAM55 vs ITA306	RAM55	1	6
RAM55 vs ITA306	RAM55	1	3
RAM55 vs ITA306	RAM55	1	2
RAM55 vs ITA306	RAM55	1	15
RAM55 vs ITA306	RAM55	1	33
RAM55 vs ITA306	RAM55	1	5
RAM55 vs ITA306	RAM55	1	115
RAM55 vs ITA306	ITA306	1	2
RAM55 vs ITA306	ITA306	1	33
RAM55 vs ITA306	ITA306	1	3
RAM55 vs ITA306	ITA306	1	1
RAM55 vs ITA306	ITA306	1	9
RAM55 vs ITA306	ITA306	1	72
RAM55 vs ITA306	ITA306	1	16
RAM55 vs ITA306	ITA306	1	42
RAM55 vs ITA306	ITA306	1	165
RAM55 vs ITA306	ITA306	1	4
RAM55 vs ITA306	ITA306	1	14
RAM55 vs ITA306	ITA306	1	37
RAM55 vs ITA306	ITA306	1	8
RAM55 vs ITA306	ITA306	1	135
RAM55 vs ITA306	ITA306	1	11
RAM55 vs ITA306	ITA306	1	53
RAM55 vs ITA306	ITA306	1	6
RAM55 vs ITA306	ITA306	1	5
RAM55 vs ITA306	ITA306	1	29
RAM55 vs ITA306	ITA306	1	17
RAM55 vs ITA306	ITA306	1	12
RAM55 vs ITA306	ITA306	1	8
RAM55 vs ITA306	ITA306	1	82
RAM55 vs ITA306	ITA306	1	34
RAM55 vs ITA306	ITA306	1	24
RAM55 vs ITA306	ITA306	1	2
RAM55 vs ITA306	ITA306	1	8
RAM55 vs ITA306	ITA306	1	187
RAM55 vs ITA306	ITA306	1	36
RAM55 vs ITA306	ITA306	1	46
RAM55 vs ITA306	ITA306	1	11
RAM55 vs ITA306	ITA306	1	5

RAM55 vs ITA306	ITA306	1	142
RAM55 vs ITA306	ITA306	1	87
RAM55 vs ITA306	ITA306	1	5
RAM55 vs ITA306	ITA306	1	6
RAM55 vs ITA306	ITA306	1	5
RAM55 vs ITA306	ITA306	1	9
RAM55 vs ITA306	ITA306	1	91
RAM55 vs ITA306	ITA306	1	27
RAM55 vs ITA306	ITA306	1	8
RAM55 vs ITA306	ITA306	1	6
RAM55 vs ITA306	ITA306	1	3
RAM55 vs ITA306	ITA306	1	78
RAM55 vs ITA306	ITA306	1	16
RAM55 vs ITA306	ITA306	1	55
RAM55 vs ITA306	ITA306	1	51
WAB56-104 vs ITA306	WAB56-104	1	9
WAB56-104 vs ITA306	WAB56-104	1	3
WAB56-104 vs ITA306	WAB56-104	1	18
WAB56-104 vs ITA306	WAB56-104	1	245
WAB56-104 vs ITA306	WAB56-104	1	246
WAB56-104 vs ITA306	WAB56-104	1	21
WAB56-104 vs ITA306	WAB56-104	1	73
WAB56-104 vs ITA306	WAB56-104	1	248
WAB56-104 vs ITA306	WAB56-104	1	25
WAB56-104 vs ITA306	WAB56-104	1	18
WAB56-104 vs ITA306	WAB56-104	1	24
WAB56-104 vs ITA306	WAB56-104	1	32
WAB56-104 vs ITA306	WAB56-104	1	27
WAB56-104 vs ITA306	WAB56-104	1	5
WAB56-104 vs ITA306	WAB56-104	1	149
WAB56-104 vs ITA306	WAB56-104	1	15
WAB56-104 vs ITA306	WAB56-104	1	31
WAB56-104 vs ITA306	WAB56-104	1	16
WAB56-104 vs ITA306	WAB56-104	1	17
WAB56-104 vs ITA306	WAB56-104	1	75
WAB56-104 vs ITA306	WAB56-104	1	57
WAB56-104 vs ITA306	WAB56-104	1	4
WAB56-104 vs ITA306	WAB56-104	1	35
WAB56-104 vs ITA306	WAB56-104	1	48
WAB56-104 vs ITA306	WAB56-104	1	28
WAB56-104 vs ITA306	WAB56-104	1	28
WAB56-104 vs ITA306	WAB56-104	1	4
WAB56-104 vs ITA306	WAB56-104	1	68
WAB56-104 vs ITA306	WAB56-104	1	9
WAB56-104 vs ITA306	WAB56-104	1	7
WAB56-104 vs ITA306	WAB56-104	1	26
WAB56-104 vs ITA306	WAB56-104	1	17

WAB56-104 vs ITA306	WAB56-104	1	128
WAB56-104 vs ITA306	WAB56-104	1	15
WAB56-104 vs ITA306	WAB56-104	1	2
WAB56-104 vs ITA306	WAB56-104	1	13
WAB56-104 vs ITA306	WAB56-104	1	7
WAB56-104 vs ITA306	WAB56-104	1	6
WAB56-104 vs ITA306	WAB56-104	1	31
WAB56-104 vs ITA306	WAB56-104	1	4
WAB56-104 vs ITA306	ITA306	1	3
WAB56-104 vs ITA306	ITA306	1	2
WAB56-104 vs ITA306	ITA306	1	27
WAB56-104 vs ITA306	ITA306	1	5
WAB56-104 vs ITA306	ITA306	1	4
WAB56-104 vs ITA306	ITA306	1	9
WAB56-104 vs ITA306	ITA306	1	87
WAB56-104 vs ITA306	ITA306	1	6
WAB56-104 vs ITA306	ITA306	1	12
WAB56-104 vs ITA306	ITA306	1	26
WAB56-104 vs ITA306	ITA306	1	12
WAB56-104 vs ITA306	ITA306	1	135
WAB56-104 vs ITA306	ITA306	1	34
WAB56-104 vs ITA306	ITA306	1	69
WAB56-104 vs ITA306	ITA306	1	1
WAB56-104 vs ITA306	ITA306	1	6
WAB56-104 vs ITA306	ITA306	1	6
WAB56-104 vs ITA306	ITA306	1	3
WAB56-104 vs ITA306	ITA306	1	11
WAB56-104 vs ITA306	ITA306	1	78
WAB56-104 vs ITA306	ITA306	1	67
WAB56-104 vs ITA306	ITA306	1	45
WAB56-104 vs ITA306	ITA306	1	2
WAB56-104 vs ITA306	ITA306	1	68
WAB56-104 vs ITA306	ITA306	1	8
WAB56-104 vs ITA306	ITA306	1	55
WAB56-104 vs ITA306	ITA306	1	76
WAB56-104 vs ITA306	ITA306	1	133
WAB56-104 vs ITA306	ITA306	1	22
WAB56-104 vs ITA306	ITA306	1	15
WAB56-104 vs ITA306	ITA306	1	78
WAB56-104 vs ITA306	ITA306	1	4
WAB56-104 vs ITA306	ITA306	1	18
WAB56-104 vs ITA306	ITA306	1	47
WAB56-104 vs ITA306	ITA306	1	92
WAB56-104 vs ITA306	ITA306	1	111
WAB56-104 vs ITA306	ITA306	1	22
WAB56-104 vs ITA306	ITA306	1	6
WAB56-104 vs RAM55	WAB56-104	1	5

WAB56-104 vs RAM55	WAB56-104	1	15
WAB56-104 vs RAM55	WAB56-104	1	17
WAB56-104 vs RAM55	WAB56-104	1	283
WAB56-104 vs RAM55	WAB56-104	1	114
WAB56-104 vs RAM55	WAB56-104	1	82
WAB56-104 vs RAM55	WAB56-104	1	24
WAB56-104 vs RAM55	WAB56-104	1	2
WAB56-104 vs RAM55	WAB56-104	1	44
WAB56-104 vs RAM55	WAB56-104	1	46
WAB56-104 vs RAM55	WAB56-104	1	4
WAB56-104 vs RAM55	WAB56-104	1	22
WAB56-104 vs RAM55	WAB56-104	1	138
WAB56-104 vs RAM55	WAB56-104	1	31
WAB56-104 vs RAM55	WAB56-104	1	81
WAB56-104 vs RAM55	WAB56-104	1	5
WAB56-104 vs RAM55	WAB56-104	1	11
WAB56-104 vs RAM55	WAB56-104	1	24
WAB56-104 vs RAM55	WAB56-104	1	4
WAB56-104 vs RAM55	WAB56-104	1	68
WAB56-104 vs RAM55	WAB56-104	1	34
WAB56-104 vs RAM55	WAB56-104	1	59
WAB56-104 vs RAM55	WAB56-104	1	237
WAB56-104 vs RAM55	WAB56-104	1	2
WAB56-104 vs RAM55	WAB56-104	1	73
WAB56-104 vs RAM55	WAB56-104	1	85
WAB56-104 vs RAM55	WAB56-104	1	3
WAB56-104 vs RAM55	WAB56-104	1	77
WAB56-104 vs RAM55	WAB56-104	1	294
WAB56-104 vs RAM55	WAB56-104	1	42
WAB56-104 vs RAM55	WAB56-104	1	66
WAB56-104 vs RAM55	WAB56-104	1	3
WAB56-104 vs RAM55	WAB56-104	1	95
WAB56-104 vs RAM55	WAB56-104	1	92
WAB56-104 vs RAM55	WAB56-104	1	7
WAB56-104 vs RAM55	WAB56-104	1	53
WAB56-104 vs RAM55	WAB56-104	1	7
WAB56-104 vs RAM55	WAB56-104	1	15
WAB56-104 vs RAM55	WAB56-104	1	13
WAB56-104 vs RAM55	WAB56-104	1	56
WAB56-104 vs RAM55	WAB56-104	1	56
WAB56-104 vs RAM55	WAB56-104	1	96
WAB56-104 vs RAM55	WAB56-104	1	94
WAB56-104 vs RAM55	WAB56-104	1	7
WAB56-104 vs RAM55	WAB56-104	1	6
WAB56-104 vs RAM55	WAB56-104	1	58
WAB56-104 vs RAM55	WAB56-104	1	9
WAB56-104 vs RAM55	WAB56-104	1	57

WAB56-104 vs RAM55	WAB56-104	1	29
WAB56-104 vs RAM55	RAM55	1	165
WAB56-104 vs RAM55	RAM55	1	34
WAB56-104 vs RAM55	RAM55	1	8
WAB56-104 vs RAM55	RAM55	1	28
WAB56-104 vs RAM55	RAM55	1	4
WAB56-104 vs RAM55	RAM55	1	49
WAB56-104 vs RAM55	RAM55	1	28
WAB56-104 vs RAM55	RAM55	1	19
WAB56-104 vs RAM55	RAM55	1	5
WAB56-104 vs RAM55	RAM55	1	54
WAB56-104 vs RAM55	RAM55	1	6
WAB56-104 vs RAM55	RAM55	1	42
WAB56-104 vs RAM55	RAM55	1	7
WAB56-104 vs RAM55	RAM55	1	27
WAB56-104 vs RAM55	RAM55	1	7
WAB56-104 vs RAM55	RAM55	1	3
WAB56-104 vs RAM55	RAM55	1	88
WAB56-104 vs RAM55	RAM55	1	211
WAB56-104 vs RAM55	RAM55	1	192
WAB56-104 vs RAM55	RAM55	1	96
WAB56-104 vs RAM55	RAM55	1	47
WAB56-104 vs RAM55	RAM55	1	152
CG14 vs RAM55	CG14	1	81
CG14 vs RAM55	CG14	1	37
CG14 vs RAM55	CG14	1	101
CG14 vs RAM55	CG14	1	60
CG14 vs RAM55	CG14	1	40
CG14 vs RAM55	CG14	1	71
CG14 vs RAM55	CG14	1	25
CG14 vs RAM55	CG14	1	118
CG14 vs RAM55	CG14	1	67
CG14 vs RAM55	CG14	1	65
CG14 vs RAM55	CG14	1	23
CG14 vs RAM55	CG14	1	70
CG14 vs RAM55	CG14	1	76
CG14 vs RAM55	CG14	1	55
CG14 vs RAM55	CG14	1	26
CG14 vs RAM55	CG14	1	37
CG14 vs RAM55	CG14	1	29
CG14 vs RAM55	CG14	1	50
CG14 vs RAM55	CG14	1	29
CG14 vs RAM55	CG14	1	50
CG14 vs RAM55	CG14	1	69
CG14 vs RAM55	CG14	1	47
CG14 vs RAM55	CG14	1	42
CG14 vs RAM55	CG14	1	29

CG14 vs RAM55	CG14	1	56
CG14 vs RAM55	CG14	1	34
CG14 vs RAM55	CG14	1	29
CG14 vs RAM55	CG14	1	69
CG14 vs RAM55	CG14	1	99
CG14 vs RAM55	CG14	1	119
CG14 vs RAM55	CG14	1	58
CG14 vs RAM55	CG14	1	56
CG14 vs RAM55	CG14	1	71
CG14 vs RAM55	CG14	1	67
CG14 vs RAM55	CG14	1	50
CG14 vs RAM55	CG14	1	57
CG14 vs RAM55	CG14	1	121
CG14 vs RAM55	RAM55	1	114
CG14 vs RAM55	RAM55	1	33
CG14 vs RAM55	RAM55	1	45
CG14 vs RAM55	RAM55	1	55
CG14 vs RAM55	RAM55	1	82
CG14 vs RAM55	RAM55	1	53
CG14 vs RAM55	RAM55	1	54
CG14 vs RAM55	RAM55	1	27
CG14 vs RAM55	RAM55	1	39
CG14 vs RAM55	RAM55	1	59
CG14 vs RAM55	RAM55	1	24
CG14 vs RAM55	RAM55	1	46
CG14 vs RAM55	RAM55	1	80
CG14 vs RAM55	RAM55	1	128
CG14 vs RAM55	RAM55	1	29
CG14 vs RAM55	RAM55	1	70
CG14 vs RAM55	RAM55	1	141
CG14 vs RAM55	RAM55	1	85
CG14 vs RAM55	RAM55	1	93
CG14 vs WAB56-104	CG14	1	83
CG14 vs WAB56-104	CG14	1	84
CG14 vs WAB56-104	CG14	1	59
CG14 vs WAB56-104	CG14	1	72
CG14 vs WAB56-104	CG14	1	64
CG14 vs WAB56-104	CG14	1	57
CG14 vs WAB56-104	CG14	1	77
CG14 vs WAB56-104	CG14	1	39
CG14 vs WAB56-104	CG14	1	55
CG14 vs WAB56-104	CG14	1	62
CG14 vs WAB56-104	CG14	1	42
CG14 vs WAB56-104	CG14	1	51
CG14 vs WAB56-104	CG14	1	47
CG14 vs WAB56-104	CG14	1	73
CG14 vs WAB56-104	CG14	1	112

CG14 vs WAB56-104	CG14	1	77
CG14 vs WAB56-104	CG14	1	42
CG14 vs WAB56-104	CG14	1	110
CG14 vs WAB56-104	CG14	1	64
CG14 vs WAB56-104	CG14	1	59
CG14 vs WAB56-104	CG14	1	78
CG14 vs WAB56-104	CG14	1	93
CG14 vs WAB56-104	CG14	1	57
CG14 vs WAB56-104	CG14	1	66
CG14 vs WAB56-104	CG14	1	105
CG14 vs WAB56-104	CG14	1	90
CG14 vs WAB56-104	CG14	1	96
CG14 vs WAB56-104	CG14	1	128
CG14 vs WAB56-104	CG14	1	78
CG14 vs WAB56-104	CG14	1	76
CG14 vs WAB56-104	CG14	1	78
CG14 vs WAB56-104	CG14	1	130
CG14 vs WAB56-104	CG14	1	82
CG14 vs WAB56-104	WAB56-104	1	163
CG14 vs WAB56-104	WAB56-104	1	50
CG14 vs WAB56-104	WAB56-104	1	59
CG14 vs WAB56-104	WAB56-104	1	68
CG14 vs WAB56-104	WAB56-104	1	45
CG14 vs WAB56-104	WAB56-104	1	62
CG14 vs WAB56-104	WAB56-104	1	35
CG14 vs WAB56-104	WAB56-104	1	61
CG14 vs WAB56-104	WAB56-104	1	106
CG14 vs WAB56-104	WAB56-104	1	53
CG14 vs WAB56-104	WAB56-104	1	84
CG14 vs WAB56-104	WAB56-104	1	91
CG14 vs WAB56-104	WAB56-104	1	68
CG14 vs WAB56-104	WAB56-104	1	59
CG14 vs WAB56-104	WAB56-104	1	68
CG14 vs WAB56-104	WAB56-104	1	37
CG14 vs WAB56-104	WAB56-104	1	50
CG14 vs WAB56-104	WAB56-104	1	142
CG14 vs WAB56-104	WAB56-104	1	88
CG14 vs WAB56-104	WAB56-104	1	71
CG14 vs WAB56-104	WAB56-104	1	70
CG14 vs WAB56-104	WAB56-104	1	76
CG14 vs ITA306	CG14	1	291
CG14 vs ITA306	CG14	1	91
CG14 vs ITA306	CG14	1	163
CG14 vs ITA306	CG14	1	30
CG14 vs ITA306	CG14	1	55
CG14 vs ITA306	CG14	1	25
CG14 vs ITA306	CG14	1	213

CG14 vs ITA306	CG14	1	35
CG14 vs ITA306	CG14	1	27
CG14 vs ITA306	CG14	1	175
CG14 vs ITA306	CG14	1	31
CG14 vs ITA306	CG14	1	75
CG14 vs ITA306	CG14	1	40
CG14 vs ITA306	CG14	1	91
CG14 vs ITA306	CG14	1	123
CG14 vs ITA306	CG14	1	58
CG14 vs ITA306	CG14	1	87
CG14 vs ITA306	CG14	1	34
CG14 vs ITA306	CG14	1	128
CG14 vs ITA306	CG14	1	49
CG14 vs ITA306	CG14	1	88
CG14 vs ITA306	CG14	1	145
CG14 vs ITA306	CG14	1	105
CG14 vs ITA306	CG14	1	85
CG14 vs ITA306	CG14	1	69
CG14 vs ITA306	CG14	1	121
CG14 vs ITA306	CG14	1	72
CG14 vs ITA306	CG14	1	53
CG14 vs ITA306	CG14	1	49
CG14 vs ITA306	CG14	1	76
CG14 vs ITA306	CG14	1	93
CG14 vs ITA306	CG14	1	61
CG14 vs ITA306	CG14	1	61
CG14 vs ITA306	CG14	1	140
CG14 vs ITA306	CG14	1	112
CG14 vs ITA306	ITA306	1	211
CG14 vs ITA306	ITA306	1	105
CG14 vs ITA306	ITA306	1	49
CG14 vs ITA306	ITA306	1	90
CG14 vs ITA306	ITA306	1	155
CG14 vs ITA306	ITA306	1	33
CG14 vs ITA306	ITA306	1	53
CG14 vs ITA306	ITA306	1	56
CG14 vs ITA306	ITA306	1	70
CG14 vs ITA306	ITA306	1	70
CG14 vs ITA306	ITA306	1	65
CG14 vs ITA306	ITA306	1	46
CG14 vs ITA306	ITA306	1	73
CG14 vs ITA306	ITA306	1	78
CG14 vs ITA306	ITA306	1	122
CG14 vs ITA306	ITA306	1	82
CG14 vs ITA306	ITA306	1	189
CG14 vs ITA306	ITA306	1	51
CG14 vs ITA306	ITA306	1	76

CG14 vs ITA306	ITA306	1	118
CG14 vs ITA306	ITA306	1	44
CG14 vs ITA306	ITA306	1	31
CG14 vs ITA306	ITA306	1	71
CG14 vs ITA306	ITA306	1	88
CG14 vs ITA306	ITA306	1	112
CG14 vs ITA306	ITA306	1	133
CG14 vs ITA306	ITA306	1	124
CG14 vs ITA306	ITA306	1	67
CG14 vs ITA306	ITA306	1	53
CG14 vs ITA306	ITA306	1	47
CG14 vs ITA306	ITA306	1	58
CG14 vs ITA306	ITA306	1	37
CG14 vs ITA306	ITA306	1	90
CG14 vs ITA306	ITA306	1	141
CG14 vs ITA306	ITA306	1	105
CG14 vs ITA306	ITA306	1	43
CG14 vs TOG5681	CG14	1	65
CG14 vs TOG5681	CG14	1	70
CG14 vs TOG5681	CG14	1	36
CG14 vs TOG5681	CG14	1	71
CG14 vs TOG5681	CG14	1	84
CG14 vs TOG5681	CG14	1	92
CG14 vs TOG5681	CG14	1	66
CG14 vs TOG5681	CG14	1	53
CG14 vs TOG5681	CG14	1	47
CG14 vs TOG5681	CG14	1	61
CG14 vs TOG5681	CG14	1	58
CG14 vs TOG5681	CG14	1	96
CG14 vs TOG5681	CG14	1	51
CG14 vs TOG5681	CG14	1	77
CG14 vs TOG5681	CG14	1	69
CG14 vs TOG5681	CG14	1	127
CG14 vs TOG5681	CG14	1	63
CG14 vs TOG5681	CG14	1	73
CG14 vs TOG5681	CG14	1	151
CG14 vs TOG5681	CG14	1	81
CG14 vs TOG5681	CG14	1	75
CG14 vs TOG5681	CG14	1	168
CG14 vs TOG5681	CG14	1	64
CG14 vs TOG5681	CG14	1	72
CG14 vs TOG5681	CG14	1	87
CG14 vs TOG5681	CG14	1	120
CG14 vs TOG5681	CG14	1	85
CG14 vs TOG5681	CG14	1	158
CG14 vs TOG5681	CG14	1	109
CG14 vs TOG5681	CG14	1	51

CG14 vs TOG5681	CG14	1	128
CG14 vs TOG5681	CG14	1	35
CG14 vs TOG5681	CG14	1	74
CG14 vs TOG5681	CG14	1	63
CG14 vs TOG5681	CG14	1	127
CG14 vs TOG5681	CG14	1	80
CG14 vs TOG5681	CG14	1	49
CG14 vs TOG5681	CG14	1	67
CG14 vs TOG5681	CG14	1	91
CG14 vs TOG5681	TOG5681	1	121
CG14 vs TOG5681	TOG5681	1	80
CG14 vs TOG5681	TOG5681	1	150
CG14 vs TOG5681	TOG5681	1	79
CG14 vs TOG5681	TOG5681	1	76
CG14 vs TOG5681	TOG5681	1	68
CG14 vs TOG5681	TOG5681	1	65
CG14 vs TOG5681	TOG5681	1	45
CG14 vs TOG5681	TOG5681	1	62
CG14 vs TOG5681	TOG5681	1	111
CG14 vs TOG5681	TOG5681	1	154
CG14 vs TOG5681	TOG5681	1	38
CG14 vs TOG5681	TOG5681	1	100
CG14 vs TOG5681	TOG5681	1	83
CG14 vs TOG5681	TOG5681	1	85
CG14 vs TOG5681	TOG5681	1	76
CG14 vs TOG5681	TOG5681	1	79
CG14 vs TOG5681	TOG5681	1	61
CG14 vs TOG5681	TOG5681	1	87
CG14 vs TOG5681	TOG5681	1	78
CG14 vs TOG5681	TOG5681	1	161
ITA306 vs TOG5681	ITA306	1	68
ITA306 vs TOG5681	ITA306	1	45
ITA306 vs TOG5681	ITA306	1	72
ITA306 vs TOG5681	ITA306	1	84
ITA306 vs TOG5681	ITA306	1	91
ITA306 vs TOG5681	ITA306	1	123
ITA306 vs TOG5681	ITA306	1	65
ITA306 vs TOG5681	ITA306	1	49
ITA306 vs TOG5681	ITA306	1	106
ITA306 vs TOG5681	ITA306	1	59
ITA306 vs TOG5681	ITA306	1	55
ITA306 vs TOG5681	ITA306	1	73
ITA306 vs TOG5681	ITA306	1	110
ITA306 vs TOG5681	ITA306	1	77
ITA306 vs TOG5681	ITA306	1	62
ITA306 vs TOG5681	ITA306	1	125
ITA306 vs TOG5681	ITA306	1	62

ITA306 vs TOG5681	ITA306	1	37
ITA306 vs TOG5681	ITA306	1	78
ITA306 vs TOG5681	ITA306	1	92
ITA306 vs TOG5681	ITA306	1	102
ITA306 vs TOG5681	ITA306	1	67
ITA306 vs TOG5681	ITA306	1	107
ITA306 vs TOG5681	ITA306	1	61
ITA306 vs TOG5681	ITA306	1	93
ITA306 vs TOG5681	ITA306	1	183
ITA306 vs TOG5681	ITA306	1	130
ITA306 vs TOG5681	ITA306	1	92
ITA306 vs TOG5681	ITA306	1	58
ITA306 vs TOG5681	ITA306	1	102
ITA306 vs TOG5681	ITA306	1	51
ITA306 vs TOG5681	ITA306	1	104
ITA306 vs TOG5681	ITA306	1	129
ITA306 vs TOG5681	ITA306	1	88
ITA306 vs TOG5681	ITA306	1	79
ITA306 vs TOG5681	ITA306	1	103
ITA306 vs TOG5681	ITA306	1	125
ITA306 vs TOG5681	TOG5681	1	57
ITA306 vs TOG5681	TOG5681	1	80
ITA306 vs TOG5681	TOG5681	1	101
ITA306 vs TOG5681	TOG5681	1	70
ITA306 vs TOG5681	TOG5681	1	114
ITA306 vs TOG5681	TOG5681	1	134
ITA306 vs TOG5681	TOG5681	1	121
ITA306 vs TOG5681	TOG5681	1	86
ITA306 vs TOG5681	TOG5681	1	84
ITA306 vs TOG5681	TOG5681	1	129
ITA306 vs TOG5681	TOG5681	1	87
ITA306 vs TOG5681	TOG5681	1	43
ITA306 vs TOG5681	TOG5681	1	39
ITA306 vs TOG5681	TOG5681	1	85
ITA306 vs TOG5681	TOG5681	1	60
ITA306 vs TOG5681	TOG5681	1	83
ITA306 vs TOG5681	TOG5681	1	44
ITA306 vs TOG5681	TOG5681	1	90
ITA306 vs TOG5681	TOG5681	1	71
ITA306 vs TOG5681	TOG5681	1	81
ITA306 vs TOG5681	TOG5681	1	92
ITA306 vs TOG5681	TOG5681	1	99
ITA306 vs TOG5681	TOG5681	1	67
TOG5681 vs RAM55	TOG5681	1	70
TOG5681 vs RAM55	TOG5681	1	78
TOG5681 vs RAM55	TOG5681	1	63
TOG5681 vs RAM55	TOG5681	1	48

TOG5681 vs RAM55	TOG5681	1	72
TOG5681 vs RAM55	TOG5681	1	134
TOG5681 vs RAM55	TOG5681	1	109
TOG5681 vs RAM55	TOG5681	1	97
TOG5681 vs RAM55	TOG5681	1	110
TOG5681 vs RAM55	TOG5681	1	161
TOG5681 vs RAM55	TOG5681	1	105
TOG5681 vs RAM55	TOG5681	1	100
TOG5681 vs RAM55	TOG5681	1	80
TOG5681 vs RAM55	TOG5681	1	43
TOG5681 vs RAM55	TOG5681	1	96
TOG5681 vs RAM55	TOG5681	1	77
TOG5681 vs RAM55	TOG5681	1	84
TOG5681 vs RAM55	TOG5681	1	85
TOG5681 vs RAM55	TOG5681	1	58
TOG5681 vs RAM55	TOG5681	1	88
TOG5681 vs RAM55	TOG5681	1	61
TOG5681 vs RAM55	TOG5681	1	141
TOG5681 vs RAM55	TOG5681	1	86
TOG5681 vs RAM55	TOG5681	1	131
TOG5681 vs RAM55	TOG5681	1	49
TOG5681 vs RAM55	TOG5681	1	103
TOG5681 vs RAM55	TOG5681	1	52
TOG5681 vs RAM55	TOG5681	1	75
TOG5681 vs RAM55	TOG5681	1	112
TOG5681 vs RAM55	TOG5681	1	69
TOG5681 vs RAM55	TOG5681	1	128
TOG5681 vs RAM55	TOG5681	1	88
TOG5681 vs RAM55	TOG5681	1	45
TOG5681 vs RAM55	TOG5681	1	113
TOG5681 vs RAM55	TOG5681	1	106
TOG5681 vs RAM55	RAM55	1	125
TOG5681 vs RAM55	RAM55	1	51
TOG5681 vs RAM55	RAM55	1	82
TOG5681 vs RAM55	RAM55	1	121
TOG5681 vs RAM55	RAM55	1	90
TOG5681 vs RAM55	RAM55	1	92
TOG5681 vs RAM55	RAM55	1	86
TOG5681 vs RAM55	RAM55	1	112
TOG5681 vs RAM55	RAM55	1	127
TOG5681 vs RAM55	RAM55	1	141
TOG5681 vs RAM55	RAM55	1	64
TOG5681 vs RAM55	RAM55	1	103
TOG5681 vs RAM55	RAM55	1	100
TOG5681 vs RAM55	RAM55	1	133
TOG5681 vs RAM55	RAM55	1	62
TOG5681 vs RAM55	RAM55	1	90

TOG5681 vs RAM55	RAM55	1	78
TOG5681 vs RAM55	RAM55	1	89
TOG5681 vs RAM55	RAM55	1	136
TOG5681 vs RAM55	RAM55	1	85
TOG5681 vs RAM55	RAM55	1	99
TOG5681 vs RAM55	RAM55	1	101
TOG5681 vs RAM55	RAM55	1	95
TOG5681 vs RAM55	RAM55	1	120
TOG5681 vs RAM55	RAM55	1	117
CG14 vs NERICA1	CG14	1	51
CG14 vs NERICA1	CG14	1	63
CG14 vs NERICA1	CG14	1	80
CG14 vs NERICA1	CG14	1	85
CG14 vs NERICA1	CG14	1	79
CG14 vs NERICA1	CG14	1	121
CG14 vs NERICA1	CG14	1	88
CG14 vs NERICA1	CG14	1	98
CG14 vs NERICA1	CG14	1	66
CG14 vs NERICA1	CG14	1	77
CG14 vs NERICA1	CG14	1	50
CG14 vs NERICA1	CG14	1	61
CG14 vs NERICA1	CG14	1	58
CG14 vs NERICA1	CG14	1	97
CG14 vs NERICA1	CG14	1	90
CG14 vs NERICA1	CG14	1	102
CG14 vs NERICA1	CG14	1	105
CG14 vs NERICA1	CG14	1	85
CG14 vs NERICA1	CG14	1	50
CG14 vs NERICA1	CG14	1	81
CG14 vs NERICA1	CG14	1	68
CG14 vs NERICA1	CG14	1	90
CG14 vs NERICA1	CG14	1	69
CG14 vs NERICA1	CG14	1	119
CG14 vs NERICA1	CG14	1	72
CG14 vs NERICA1	CG14	1	91
CG14 vs NERICA1	CG14	1	125
CG14 vs NERICA1	CG14	1	161
CG14 vs NERICA1	CG14	1	95
CG14 vs NERICA1	CG14	1	89
CG14 vs NERICA1	CG14	1	121
CG14 vs NERICA1	CG14	1	108
CG14 vs NERICA1	CG14	1	131
CG14 vs NERICA1	CG14	1	99
CG14 vs NERICA1	NERICA1	1	70
CG14 vs NERICA1	NERICA1	1	97
CG14 vs NERICA1	NERICA1	1	66
CG14 vs NERICA1	NERICA1	1	101

CG14 vs NERICA1	NERICA1	1	72
CG14 vs NERICA1	NERICA1	1	136
CG14 vs NERICA1	NERICA1	1	62
CG14 vs NERICA1	NERICA1	1	48
CG14 vs NERICA1	NERICA1	1	70
CG14 vs NERICA1	NERICA1	1	82
CG14 vs NERICA1	NERICA1	1	124
CG14 vs NERICA1	NERICA1	1	73
CG14 vs NERICA1	NERICA1	1	91
CG14 vs NERICA1	NERICA1	1	75
CG14 vs NERICA1	NERICA1	1	100
CG14 vs NERICA1	NERICA1	1	123
CG14 vs NERICA1	NERICA1	1	83
CG14 vs NERICA1	NERICA1	1	126
CG14 vs NERICA1	NERICA1	1	96
CG14 vs NERICA1	NERICA1	1	108
CG14 vs NERICA1	NERICA1	1	67
CG14 vs NERICA1	NERICA1	1	64
CG14 vs NERICA1	NERICA1	1	114
CG14 vs NERICA1	NERICA1	1	86
CG14 vs NERICA1	NERICA1	1	119
CG14 vs NERICA8	CG14	1	73
CG14 vs NERICA8	CG14	1	95
CG14 vs NERICA8	CG14	1	84
CG14 vs NERICA8	CG14	1	122
CG14 vs NERICA8	CG14	1	67
CG14 vs NERICA8	CG14	1	108
CG14 vs NERICA8	CG14	1	50
CG14 vs NERICA8	CG14	1	70
CG14 vs NERICA8	CG14	1	125
CG14 vs NERICA8	CG14	1	87
CG14 vs NERICA8	CG14	1	105
CG14 vs NERICA8	CG14	1	65
CG14 vs NERICA8	CG14	1	63
CG14 vs NERICA8	CG14	1	115
CG14 vs NERICA8	CG14	1	45
CG14 vs NERICA8	CG14	1	104
CG14 vs NERICA8	CG14	1	59
CG14 vs NERICA8	CG14	1	140
CG14 vs NERICA8	CG14	1	72
CG14 vs NERICA8	CG14	1	92
CG14 vs NERICA8	CG14	1	109
CG14 vs NERICA8	CG14	1	118
CG14 vs NERICA8	CG14	1	81
CG14 vs NERICA8	CG14	1	108
CG14 vs NERICA8	CG14	1	78
CG14 vs NERICA8	CG14	1	125

CG14 vs NERICA8	CG14	1	98
CG14 vs NERICA8	CG14	1	87
CG14 vs NERICA8	CG14	1	82
CG14 vs NERICA8	CG14	1	104
CG14 vs NERICA8	CG14	1	132
CG14 vs NERICA8	CG14	1	90
CG14 vs NERICA8	CG14	1	97
CG14 vs NERICA8	CG14	1	121
CG14 vs NERICA8	CG14	1	97
CG14 vs NERICA8	NERICA8	1	81
CG14 vs NERICA8	NERICA8	1	100
CG14 vs NERICA8	NERICA8	1	83
CG14 vs NERICA8	NERICA8	1	90
CG14 vs NERICA8	NERICA8	1	64
CG14 vs NERICA8	NERICA8	1	76
CG14 vs NERICA8	NERICA8	1	114
CG14 vs NERICA8	NERICA8	1	88
CG14 vs NERICA8	NERICA8	1	58
CG14 vs NERICA8	NERICA8	1	98
CG14 vs NERICA8	NERICA8	1	131
CG14 vs NERICA8	NERICA8	1	68
CG14 vs NERICA8	NERICA8	1	74
CG14 vs NERICA8	NERICA8	1	85
CG14 vs NERICA8	NERICA8	1	106
CG14 vs NERICA8	NERICA8	1	124
CG14 vs NERICA8	NERICA8	1	93
CG14 vs NERICA8	NERICA8	1	83
CG14 vs NERICA8	NERICA8	1	103
CG14 vs NERICA8	NERICA8	1	94
CG14 vs NERICA8	NERICA8	1	83
CG14 vs NERICA8	NERICA8	1	112
CG14 vs NERICA8	NERICA8	1	70
CG14 vs NERICA8	NERICA8	1	101
CG14 vs NERICA8	NERICA8	1	119
NERICA1 vs Clean air	NERICA1	1	110
NERICA1 vs Clean air	NERICA1	1	91
NERICA1 vs Clean air	NERICA1	1	71
NERICA1 vs Clean air	NERICA1	1	66
NERICA1 vs Clean air	NERICA1	1	51
NERICA1 vs Clean air	NERICA1	1	121
NERICA1 vs Clean air	NERICA1	1	69
NERICA1 vs Clean air	NERICA1	1	82
NERICA1 vs Clean air	NERICA1	1	106
NERICA1 vs Clean air	NERICA1	1	110
NERICA1 vs Clean air	NERICA1	1	72
NERICA1 vs Clean air	NERICA1	1	114
NERICA1 vs Clean air	NERICA1	1	107

NERICA1 vs Clean air	NERICA1	1	75
NERICA1 vs Clean air	NERICA1	1	87
NERICA1 vs Clean air	NERICA1	1	80
NERICA1 vs Clean air	NERICA1	1	106
NERICA1 vs Clean air	NERICA1	1	96
NERICA1 vs Clean air	NERICA1	1	64
NERICA1 vs Clean air	NERICA1	1	121
NERICA1 vs Clean air	NERICA1	1	78
NERICA1 vs Clean air	NERICA1	1	117
NERICA1 vs Clean air	NERICA1	1	49
NERICA1 vs Clean air	NERICA1	1	74
NERICA1 vs Clean air	NERICA1	1	63
NERICA1 vs Clean air	NERICA1	1	104
NERICA1 vs Clean air	NERICA1	1	89
NERICA1 vs Clean air	NERICA1	1	55
NERICA1 vs Clean air	NERICA1	1	113
NERICA1 vs Clean air	NERICA1	1	129
NERICA1 vs Clean air	NERICA1	1	102
NERICA1 vs Clean air	NERICA1	1	111
NERICA1 vs Clean air	NERICA1	1	85
NERICA1 vs Clean air	Clean air	1	105
NERICA1 vs Clean air	Clean air	1	99
NERICA1 vs Clean air	Clean air	1	100
NERICA1 vs Clean air	Clean air	1	103
NERICA1 vs Clean air	Clean air	1	62
NERICA1 vs Clean air	Clean air	1	83
NERICA1 vs Clean air	Clean air	1	79
NERICA1 vs Clean air	Clean air	1	93
NERICA1 vs Clean air	Clean air	1	125
NERICA1 vs Clean air	Clean air	1	72
NERICA1 vs Clean air	Clean air	1	70
NERICA1 vs Clean air	Clean air	1	95
NERICA1 vs Clean air	Clean air	1	101
NERICA1 vs Clean air	Clean air	1	68
NERICA1 vs Clean air	Clean air	1	114
NERICA1 vs Clean air	Clean air	1	88
NERICA1 vs Clean air	Clean air	1	126
NERICA1 vs Clean air	Clean air	1	77
NERICA1 vs Clean air	Clean air	1	150
NERICA1 vs Clean air	Clean air	1	94
NERICA1 vs Clean air	Clean air	1	95
NERICA1 vs Clean air	Clean air	1	61
NERICA1 vs Clean air	Clean air	1	68
NERICA1 vs Clean air	Clean air	1	99
NERICA1 vs Clean air	Clean air	1	161
NERICA8 vs Clean air	NERICA8	1	71
NERICA8 vs Clean air	NERICA8	1	62

NERICA8 vs Clean air	NERICA8	1	37
NERICA8 vs Clean air	NERICA8	1	49
NERICA8 vs Clean air	NERICA8	1	81
NERICA8 vs Clean air	NERICA8	1	67
NERICA8 vs Clean air	NERICA8	1	54
NERICA8 vs Clean air	NERICA8	1	77
NERICA8 vs Clean air	NERICA8	1	57
NERICA8 vs Clean air	NERICA8	1	74
NERICA8 vs Clean air	NERICA8	1	80
NERICA8 vs Clean air	NERICA8	1	69
NERICA8 vs Clean air	NERICA8	1	84
NERICA8 vs Clean air	NERICA8	1	48
NERICA8 vs Clean air	NERICA8	1	103
NERICA8 vs Clean air	NERICA8	1	121
NERICA8 vs Clean air	NERICA8	1	105
NERICA8 vs Clean air	NERICA8	1	96
NERICA8 vs Clean air	NERICA8	1	87
NERICA8 vs Clean air	NERICA8	1	104
NERICA8 vs Clean air	NERICA8	1	110
NERICA8 vs Clean air	NERICA8	1	109
NERICA8 vs Clean air	NERICA8	1	102
NERICA8 vs Clean air	NERICA8	1	107
NERICA8 vs Clean air	NERICA8	1	124
NERICA8 vs Clean air	NERICA8	1	91
NERICA8 vs Clean air	NERICA8	1	63
NERICA8 vs Clean air	NERICA8	1	46
NERICA8 vs Clean air	NERICA8	1	78
NERICA8 vs Clean air	NERICA8	1	140
NERICA8 vs Clean air	NERICA8	1	108
NERICA8 vs Clean air	NERICA8	1	133
NERICA8 vs Clean air	Clean air	1	45
NERICA8 vs Clean air	Clean air	1	65
NERICA8 vs Clean air	Clean air	1	55
NERICA8 vs Clean air	Clean air	1	73
NERICA8 vs Clean air	Clean air	1	61
NERICA8 vs Clean air	Clean air	1	92
NERICA8 vs Clean air	Clean air	1	90
NERICA8 vs Clean air	Clean air	1	101
NERICA8 vs Clean air	Clean air	1	93
NERICA8 vs Clean air	Clean air	1	86
NERICA8 vs Clean air	Clean air	1	79
NERICA8 vs Clean air	Clean air	1	111
NERICA8 vs Clean air	Clean air	1	118
NERICA8 vs Clean air	Clean air	1	124
NERICA8 vs Clean air	Clean air	1	98
NERICA8 vs Clean air	Clean air	1	70
NERICA8 vs Clean air	Clean air	1	89

NERICA8 vs Clean air	Clean air	1	52
NERICA8 vs Clean air	Clean air	1	98
NERICA8 vs Clean air	Clean air	1	75
NERICA8 vs Clean air	Clean air	1	86
NERICA8 vs Clean air	Clean air	1	116
NERICA8 vs Clean air	Clean air	1	113
NERICA8 vs Clean air	Clean air	1	92
NERICA8 vs Clean air	Clean air	1	82
NERICA8 vs Clean air	Clean air	1	105
