# Supplementary material for: Determination of Rice Accession Status Using Infochemical and Visual Cues Emitted to Sustainably Control Diopsis apicalis Dalman
Source: Insects. 2025 Jul 23;16(8):752. doi: 10.3390/insects16080752 (PMC12386945; doi:10.3390/insects16080752)
Supplement: Supplementary file 1 [file insects-16-00752-s001.zip › Table S2. WAB56-104 vs Clean air assessment.pdf]

Test N°	WAB56-104	WAB arm duration	Clean air	Duration Clean air
1	1	26	0	
2	1	22	0	
3	1	3	0	
4	0		0	
5	1	2	0	
6	0		1	16
7	1	5	0	
8	1	27	0	
9	1	82	0	
10	0		1	138
11	1	8	0	
12	1	147	0	
13	0		1	6
14	0		1	5
15	1	95	0	
16	0		0	
17	1	6	0	
18	0		1	5
19	1	18	0	
20	0		1	13
21	0		1	4
22	1	4	0	

23	1	13	0	
24	1	12	0	
25	1	6	0	
26	0		1	2
27	1	3	0	
28	1	165	0	
29	0		1	32
30	0		1	13
31	1	9	0	
32	1	42	0	
33	0		1	277
34	0		1	6
35	0		1	17
36	1	5	0	
37	1	1	0	
38	1	2	0	
39	1	5	0	
40	1	12	0	
41	1	37	0	
42	0		1	3
43	0		0	
44	0		1	86
45	1	2	0	
46	1	8	0	
47	1	99	0	
48	1	6	0	
49	1	51	0	

50	0		1	11
51	1	119	0	
52	0		1	41
53	1	3	0	
54	1	132	0	
55	1	191	0	
56	1	145	0	
57	1	227	0	
58	1	6	0	
59	0		0	
60	1	15	0	
**Total**	**39**		**17**	
Mean duration	45.15	**45.15**	39.71	**39.71**
Percentage	70		30	
Speed	2.88	2.88	3.27	3.27
Standard dev	0.460888599	79.50697438	0.383482494	37.74917218

No choice	Speed WAB arm duration	Speed Air Column conv.
0	5	
0	5.99999991	
0	4.333333333	
1		
0	65	
0		8.125
0	26	
0	4.814814815	
0	1.585365854	
0		0.942289856
0	16.25	
0	0.884353741	
0		21.66666667
0		2.6
0	1.368421526	
1		
0	21.66666667	
0		26
0	7.222222222	
0		1
0		32.5
0	32.5	

0	1	
0	1.833333333	
0	21.66666667	
0		65
0	43.33333333	
0	0.787878788	
0		4.625
0		1
0	14.44444444	
0	3.952389524	
0		0.469314794
0		21.66666667
0		7.647588235
0	26	
0	13	
0	6.5	
0	26	
0	1.833333333	
0	3.513513514	
0		43.33333333
1		
0		1.51162797
0	65	
0	16.25	
0	1.313131313	
0	21.66666667	
0	2.549196784	

0				11.81818182
0		1.924369748		
0				3.177317732
0		43.33333333		
0		0.984848485		
0		0.686282723		
0		0.896551724		
0		0.572687225		
0		21.66666667		
1				
0		8.666666667		
**4**	**#DIV/0!**		**#DIV/0!**	
0.323380833		19.89158056		
