# Supplementary material for: Determination of Rice Accession Status Using Infochemical and Visual Cues Emitted to Sustainably Control Diopsis apicalis Dalman
Source: Insects. 2025 Jul 23;16(8):752. doi: 10.3390/insects16080752 (PMC12386945; doi:10.3390/insects16080752)
Supplement: Supplementary file 1 [file insects-16-00752-s001.zip › Table S3. CG14 vs Clean air assessment.pdf]

N°	CG14	Duration CG14	Clean air	Duration clean air	No Choice
1	1	59			
2	1	82			
3					1
4	1	45			
5	1	58			
6			1	63	
7	1	35			
8	1	44			
9			1	82	
10	1	62			
11	1	36			
12	1	27			
13	1	39			
14			1	63	
15	1	46			
16	1	60			
17	1	45			
18	1	47			
19	1	30			
20					1
21	1	29			
22	1	21			
23	1	29			
24	1	19			
25	1	32			
26	1	22			
27	1	20			
28			1	27	
29					1
30	1	15			
31	1	17			
32	1	19			
33	1	27			
34			1	29	
35	1	29			
36	1	19			
37	1	35			
38	1	18			
39	1	27			
40					1
41					1
42			1	137	
43	1	20			
44			1	20	
45	1	57			
46	1	75			

	47	1	85			
	48	1	24			
	49			1	33	
	50	1	18			
	51	1	91			
	52			1	133	
	53	1	98			
	54	1	20			
	55			1	76	
	56					1
	57	1	67			
	58					1
	59	1	26			
	60	1	45			
Percentage		81.13208		18.8679245		
Total choices/ Duration		43	39.97674419	10	66.3	5
