# Supplementary material for: Determination of Rice Accession Status Using Infochemical and Visual Cues Emitted to Sustainably Control Diopsis apicalis Dalman
Source: Insects. 2025 Jul 23;16(8):752. doi: 10.3390/insects16080752 (PMC12386945; doi:10.3390/insects16080752)
Supplement: Supplementary file 1 [file insects-16-00752-s001.zip › Table S4. CG14 vs Clean air assessment.pdf]

| N° | CG14 | Duration CG14 | Clean air | Duration clean air | No Choice |
|---|---|---|---|---|---|
| 1 | 1 | 59 | | | |
| 2 | 1 | 82 | | | |
| 3 | | | | | 1 |
| 4 | 1 | 45 | | | |
| 5 | 1 | 58 | | | |
| 6 | | | 1 | 63 | |
| 7 | 1 | 35 | | | |
| 8 | 1 | 44 | | | |
| 9 | | | 1 | 82 | |
| 10 | 1 | 62 | | | |
| 11 | 1 | 36 | | | |
| 12 | 1 | 27 | | | |
| 13 | 1 | 39 | | | |
| 14 | | | 1 | 63 | |
| 15 | 1 | 46 | | | |
| 16 | 1 | 60 | | | |
| 17 | 1 | 45 | | | |
| 18 | 1 | 47 | | | |
| 19 | 1 | 30 | | | |
| 20 | | | | | 1 |
| 21 | 1 | 29 | | | |
| 22 | 1 | 21 | | | |
| 23 | 1 | 29 | | | |
| 24 | 1 | 19 | | | |
| 25 | 1 | 32 | | | |
| 26 | 1 | 22 | | | |
| 27 | 1 | 20 | | | |
| 28 | | | 1 | 27 | |
| 29 | | | | | 1 |
| 30 | 1 | 15 | | | |
| 31 | 1 | 17 | | | |
| 32 | 1 | 19 | | | |
| 33 | 1 | 27 | | | |
| 34 | | | 1 | 29 | |
| 35 | 1 | 29 | | | |
| 36 | 1 | 19 | | | |
| 37 | 1 | 35 | | | |
| 38 | 1 | 18 | | | |
| 39 | 1 | 27 | | | |
| 40 | | | | | 1 |
| 41 | | | | | 1 |
| 42 | | | 1 | 137 | |
| 43 | 1 | 20 | | | |
| 44 | | | 1 | 20 | |
| 45 | 1 | 57 | | | |
| 46 | 1 | 75 | | | |

| | | | | | | |
|---|---|---|---|---|---|---|
| | 47 | 1 | 85 | | | |
| | 48 | 1 | 24 | | | |
| | 49 | | | 1 | 33 | |
| | 50 | 1 | 18 | | | |
| | 51 | 1 | 91 | | | |
| | 52 | | | 1 | 133 | |
| | 53 | 1 | 98 | | | |
| | 54 | 1 | 20 | | | |
| | 55 | | | 1 | 76 | |
| | 56 | | | | | 1 |
| | 57 | 1 | 67 | | | |
| | 58 | | | | | 1 |
| | 59 | 1 | 26 | | | |
| | 60 | 1 | 45 | | | |
| Percentage | | 81.13208 | | 18.8679245 | | |
| Total choices/ Duration | | 43 | 39.97674419 | 10 | 66.3 | 5 |
