# Supplementary material for: Determination of Rice Accession Status Using Infochemical and Visual Cues Emitted to Sustainably Control Diopsis apicalis Dalman
Source: Insects. 2025 Jul 23;16(8):752. doi: 10.3390/insects16080752 (PMC12386945; doi:10.3390/insects16080752)
Supplement: Supplementary file 1 [file insects-16-00752-s001.zip › Table S5. WAB56-104 vs ITA306 assessment.pdf]

Test N°	WAB Column	ITA Column	No choice
1	0	1	0
2	1	0	0
3	0	1	0
4	1	0	0
5	0	1	0
6	0	1	0
7	1	0	0
8	1	0	0
9	1	0	0
10	0	1	0
11	0	1	0
12	0	0	1
13	1	0	0
14	0	1	0
15	1	0	0
16	1	0	0
17	1	0	0
18	0	1	0
19	0	1	0
20	1	0	0
21	0	0	1
22	1	0	0

23	0	1	0
24	1	0	0
25	0	1	0
26	0	1	0
27	0	1	0
28	0	1	0
29	0	1	0
30	1	0	0
31	0	1	0
32	0	1	0
33	0	1	0
34	0	1	0
35	1	0	0
36	1	0	0
37	1	0	0
38	1	0	0
39	1	0	0
40	1	0	0
41	1	0	0
42	0	1	0
43	0	1	0
44	1	0	0
45	1	0	0
46	1	0	0
47	1	0	0

48	1	0	0
49	1	0	0
50	0	1	0
51	0	1	0
52	1	0	0
53	0	1	0
54	0	1	0
55	1	0	0
56	1	0	0
57	0	1	0
58	1	0	0
59	1	0	0
60	0	1	0
61	0	1	0
62	1	0	0
63	1	0	0
64	0	1	0
65	0	1	0
66	0	1	0
67	1	0	0
68	1	0	0
69	1	0	0
70	0	1	0
71	1	0	0
72	0	1	0
73	0	1	0
74	0	1	0
75	0	1	0
76	1	0	0
77	1	0	0

78	0	1	0
79	0	1	0
80	1	0	0
**Total**	**40**	**38**	**2**
Mean duration	45.85	35.86	
Percents	51	49	
Speed	2.84	3.62	
Standard dev	0.505227924	0.505227924	0

WAB arm duration	ITA arm duration	Speed WAB arm duration
	3	
9		1.444444444
	2	
3		43.33333333
	27	
	5	
18		7.222222222
245		0.536122449
246		0.528455285
	4	
	9	
21		6.194761948
	87	
73		1.788219179
248		0.524193548
25		5.2
	6	
	12	
18		7.222222222
24		5.416666667

	26	
32		4.625
	12	
	135	
	34	
	69	
	1	
27		4.814814815
	6	
	6	
	3	
	11	
5		26
149		0.872483221
15		8.666666667
31		4.193548388
16		8.125
17		7.647588235
75		1.733333333
	78	
	67	
57		2.287175439
4		32.5
35		3.714285714
48		2.783333333

28		4.642857143
28		4.642857143
	45	
	2	
4		3.25
	68	
	8	
68		1.911764759
9		14.44444444
	55	
7		18.57142857
26		5
	76	
	133	
17		7.647588235
128		1.15625
	22	
	15	
	78	
15		8.666666667
2		65
13		1
	4	
7		18.57142857
	18	
	47	
	92	
	111	
6		21.66666667
31		4.193548388

	22		
	6		
4			32.5
		**#DIV/0!**	
**45.85**	**36.97**		
2.84	3.62		
30.36839597	40.32560492		15.79204799

Speed ITA arm duration

43.33333333

65

4.814814815

26

3.25

14.44444444

1.494252874

21.66666667

1.833333333

5

1.833333333

0.962962963

3.823529412

1.884579711

13

21.66666667

2.166666667

0.433333333

11.81818182

#VALUE!

1.942985746

2.888888889
6.5
1.911764759
16.25
2.363636364
1.715263158
0.977443692
5.99999991
8.666666667
1.666666667
3.25
7.222222222
2.765957447
1.413434783
1.171171171

5.99999991

21.66666667

#DIV/0!

5.691707948
