# Supplementary material for: Determination of Rice Accession Status Using Infochemical and Visual Cues Emitted to Sustainably Control Diopsis apicalis Dalman
Source: Insects. 2025 Jul 23;16(8):752. doi: 10.3390/insects16080752 (PMC12386945; doi:10.3390/insects16080752)
Supplement: Supplementary file 1 [file insects-16-00752-s001.zip › Table S6. RAM55 vs ITA306 assessment.pdf]

Test N°	RAM55	ITA306	No choice	RAM55 arm duration	ITA arm duration
1	0	1	0		2
2	0	1	0		33
3	0	1	0		3
4	0	1	0		1
5	0	1	0		9
6	1	0	0	4	
7	0	1	0		72
8	0	1	0		16
9	0	1	0		42
10	1	0	0	43	
11	0	1	0		165
12	0	1	0		4
13	0	1	0		14
14	1	0	0	41	
15	0	1	0		37
16	1	0	0	82	
17	1	0	0	12	
18	0	1	0		8
19	1	0	0	43	
20	0	1	0		135
21	0	1	0		11
22	1	0	0	3	

23	0	1	0		53
24	0	1	0		6
25	0	1	0		5
26	1	0	0	41	
27	0	0	1		
28	0	1	0		29
29	0	1	0		17
30	1	0	0	1	
31	0	1	0		12
32	0	1	0		8
33	0	1	0		82
34	0	1	0		34
35	1	0	0	19	
36	0	1	0		24
37	1	0	0	1	
38	0	1	0		2
39	0	1	0		8
40	1	0	0	12	
41	0	1	0		187
42	1	0	0	7	
43	1	0	0	138	
44	0	1	0		36
45	1	0	0	148	
46	0	0	1		
47	0	1	0		46

48	1	0	0	5	
49	0	1	0		11
50	0	1	0		5
51	1	0	0	18	
52	0	0	1		
53	1	0	0	22	
54	0	1	0		142
55	0	1	0		87
56	0	1	0		5
57	0	1	0		6
58	0	0	1		
59	0	1	0		5
60	0	1	0		9
61	0	1	0		91
62	1	0	0	49	
63	0	1	0		27
64	0	1	0		8
65	0	1	0		6
66	1	0	0	6	
67	0	1	0		3
68	1	0	0	3	
69	0	1	0		78
70	1	0	0	2	
71	1	0	0	15	
72	1	0	0	33	
73	0	1	0		16
74	0	1	0		55
75	0	1	0		51
76	0	0	1		
77	1	0	0	5	

78	1	0	0	115	
79	0	0	1		
80	0	1	0		1
**Total**	**26**	**48**	**6**		
Mean duratior	33.38	35.56		**33.38**	**35.56**
Percents	35	65			
Speed	3.89	3.66		3.89	3.66
Standard dev	0.470824	0.50612	0.3509312	36.32071646	42.60653315

Sped RAM55 arm duration	Speed ITA arm duration
	6.5
	3.939393939
	43.33333333
	13
	1.444444444
32.5	
	1.855555556
	8.125
	3.952389524
3.23255814	
	0.787878788
	32.5
	9.285714286
3.177317732	
	3.513513514
1.585365854	
1.833333333	
	16.25
3.23255814	
	0.962962963
	11.81818182
43.33333333	

	2.452831887
	21.66666667
	2.6
3.177317732	
	4.482758627
	1.214953271
13	
	1.833333333
	16.25
	1.585365854
	3.823529412
6.842152632	
	5.416666667
13	
	65
	16.25
1.833333333	
	0.695187166
18.57142857	
0.942289856	
	3.611111111
0.878378378	
	2.826869565

26	
	11.81818182
	26
7.222222222	
5.99999991	
	0.915492958
	1.494252874
	2.6
	2.166666667
	26
	14.44444444
	1.428571429
2.653612245	
	4.814814815
	16.25
	2.166666667
21.66666667	
	4.333333333
43.33333333	
	1.666666667
65	
8.666666667	
3.939393939	
	8.125
	2.363636364
	2.549196784
26	

	1.134347827		
			13
**#DIV/0!**		**#DIV/0!**	
	21.92924178		7.218743631
