# Supplementary material for: Determination of Rice Accession Status Using Infochemical and Visual Cues Emitted to Sustainably Control Diopsis apicalis Dalman
Source: Insects. 2025 Jul 23;16(8):752. doi: 10.3390/insects16080752 (PMC12386945; doi:10.3390/insects16080752)
Supplement: Supplementary file 1 [file insects-16-00752-s001.zip › Table S7. CG14 vs ITA306 assessment.pdf]

N°	CG14	Duration CG14	ITA306	Duration ITA306	No choice
1			1	211	
2	1	291			
3	1	91			
4			1	105	
5					1
6			1	49	
7	1	163			
8	1	30			
9	1	55			
10			1	90	
11			1	155	
12	1	25			
13					1
14					1
15	1	213			
16	1	35			
17	1	27			
18					1
19	1	175			
20	1	31			
21	1	75			
22	1	40			
23			1	33	
24			1	53	
25	1	91			
26	1	123			
27			1	56	
28			1	70	
29			1	70	
30					1
31	1	58			
32	1	87			
33	1	34			
34					1
35			1	65	
36	1	128			
37			1	46	
38			1	73	
39	1	49			
40			1	78	
41					1
42			1	122	
43			1	82	
44			1	189	
45	1	88			
46			1	51	

47			1	76	
48	1	145			
49	1	105			
50			1	118	
51	1	85			
52			1	44	
53			1	31	
54	1	69			
55	1	121			
56	1	72			
57	1	53			
58			1	71	
59	1	49			
60					1
61	1	76			
62			1	88	
63			1	112	
64					1
65			1	133	
66			1	124	
67			1	67	
68			1	53	
69	1	93			
70			1	47	
71	1	61			
72			1	58	
73	1	61			
74	1	140			
75			1	37	
76			1	90	
77			1	141	
78			1	105	
79			1	43	
80	1	112			
Total choic	35	90.02857143	36	84.33333333	9
