# Supplementary material for: Determination of Rice Accession Status Using Infochemical and Visual Cues Emitted to Sustainably Control Diopsis apicalis Dalman
Source: Insects. 2025 Jul 23;16(8):752. doi: 10.3390/insects16080752 (PMC12386945; doi:10.3390/insects16080752)
Supplement: Supplementary file 1 [file insects-16-00752-s001.zip › Table S8. WAB56-104 vs RAM55 assessment.pdf]

Test N°	WAB 56-104	WAB arm duration	RAM 55	RAM arm duration	No choice Duration
1	1	5			
2	1	15			
3	1	17			
4	1	283			
5			1	165	
6	1	114			
7	1	82			
8	1	24			
9	1	2			
10			1	34	
11			1	8	
12	1	44			
13			1	28	
14					5mn
15	1	46			
16			1	4	
17	1	4			
18	1	22			
19			1	49	
20	1	138			
21					5mn
22	1	31			

23			1	28	
24					5mn
25	1	81			
26	1	5			
27	1	11			
28			1	19	
29	1	24			
30	1	4			
31			1	5	
32	1	68			
33	1	34			
34	1	59			
35			1	54	
36					5mn
37			1	6	
38					5mn
39	1	237			
40	1	2			
41					5mn
42	1	73			
43	1	85			
44	1	3			
45	1	77			
46	1	294			
47	1	42			
48	1	66			
49			1	42	
50					5mn
51					5mn

52			1	7		
53	1	3				
54			1	27		
55	1	95				
56	1	92				
57					5mn	
58			1	7		
59			1	3		
60	1	7				
61			1	88		
62	1	53				
63	1	7				
64	1	15				
65	1	13				
66	1	56				
67	1	56				
68	1	96				
69	1	94				
70	1	7				
71	1	6				
72			1	211		
73			1	192		
74	1	58				
75	1	9				
76			1	96		
77	1	57				
78	1	29				
79			1	47		
80			1	152		
**Total**	**49**		**22**			**9**
Mean durat	56.02	**56.02**	57.82	**57.82**		
Percents	69		31			
Speed	2.32	2.32	2.25	2.25		
Standard de	#REF!	#REF!	#REF!	#REF!	#REF!	

Speed WAB arm duration	Speed RAM arm duration
26	
8.666666667	
7.647588235	
0.459363958	
	0.787878788
1.143587719	
1.585365854	
5.416666667	
6.5	
	3.823529412
	16.25
2.954545455	
	4.642857143
2.826869565	
	32.5
32.5	
5.99999991	
	2.653612245
0.942289856	
4.193548388	

	4.642857143
1.649382716	
26	
11.81818182	
	6.842152632
5.416666667	
32.5	
	26
1.911764759	
3.823529412	
2.233898358	
	2.474747474
	21.66666667
0.548523268	
6.5	
1.788219179	
1.529411765	
4.333333333	
1.688311688	
0.442176877	
3.952389524	
1.96969697	
	3.952389524

			18.57142857
	0.433333333		
			0.481481481
	1.368421526		
	1.413434783		
			18.57142857
			43.33333333
	1.857142857		
			1.477272727
	2.452831887		
	18.57142857		
	1.23895239		
	1		
	2.321428571		
	2.321428571		
	1.354166667		
	1.382978723		
	18.57142857		
	21.66666667		
			0.616113745
			0.677833333
	2.241379313		
	14.44444444		
			1.354166667
	2.287175439		
	4.482758627		
			2.765957447
			0.855263158
**#DIV/0!**		**#DIV/0!**	
#REF!		#REF!	
