# Supplementary material for: Determination of Rice Accession Status Using Infochemical and Visual Cues Emitted to Sustainably Control Diopsis apicalis Dalman
Source: Insects. 2025 Jul 23;16(8):752. doi: 10.3390/insects16080752 (PMC12386945; doi:10.3390/insects16080752)
Supplement: Supplementary file 1 [file insects-16-00752-s001.zip › Table S9. CG14 vs WAB56-104 assessment.pdf]

| N° | CG14 | Duration CG14 | WAB56-104 | Duration WAB56-104 | No Choice |
|---|---|---|---|---|---|
| 1 | 1 | 83 | | | |
| 2 | | | 1 | 163 | |
| 3 | 1 | 84 | | | |
| 4 | 1 | 59 | | | |
| 5 | 1 | 72 | | | |
| 6 | 1 | 64 | | | |
| 7 | | | | | 1 |
| 8 | | | 1 | 50 | |
| 9 | 1 | 57 | | | |
| 10 | 1 | 77 | | | |
| 11 | | | 1 | 59 | |
| 12 | 1 | 39 | | | |
| 13 | | | 1 | 68 | |
| 14 | | | 1 | 45 | |
| 15 | | | 1 | 62 | |
| 16 | 1 | 55 | | | |
| 17 | 1 | 62 | | | |
| 18 | 1 | 42 | | | |
| 19 | | | 1 | 35 | |
| 20 | 1 | 51 | | | |
| 21 | 1 | 47 | | | |
| 22 | | | | | 1 |
| 23 | 1 | 73 | | | |
| 24 | 1 | 112 | | | |
| 25 | 1 | 77 | | | |
| 26 | | | 1 | 61 | |
| 27 | 1 | 42 | | | |
| 28 | | | 1 | 106 | |
| 29 | 1 | 110 | | | |
| 30 | | | 1 | 53 | |
| 31 | | | 1 | 84 | |
| 32 | | | 1 | 91 | |
| 33 | | | 1 | 68 | |
| 34 | | | 1 | 59 | |
| 35 | 1 | 64 | | | |
| 36 | 1 | 59 | | | |
| 37 | | | | | 1 |
| 38 | | | 1 | 68 | |
| 39 | | | | | 1 |
| 40 | 1 | 78 | | | |
| 41 | | | 1 | 37 | |
| 42 | | | 1 | 50 | |
| 43 | | | 1 | 142 | |
| 44 | 1 | 93 | | | |
| 45 | 1 | 57 | | | |
| 46 | 1 | 66 | | | |

| | | | | | |
|---|---|---|---|---|---|
| 47 | 1 | 105 | | | |
| 48 | 1 | 90 | | | |
| 49 | | | 1 | 88 | |
| 50 | 1 | 96 | | | |
| 51 | | | 1 | 71 | |
| 52 | | | 1 | 70 | |
| 53 | 1 | 128 | | | |
| 54 | 1 | 78 | | | |
| 55 | | | | | 1 |
| 56 | | | 1 | 76 | |
| 57 | 1 | 76 | | | |
| 58 | 1 | 78 | | | |
| 59 | 1 | 130 | | | |
| 60 | 1 | 82 | | | |
| Percent | 60 | | 40 | | |
| Tot cho | 33 | 75.33333333 | 22 | 73 | 5 |
